# Bridging Smart Nanosystems with Clinically Relevant Models and Advanced Imaging for Precision Drug Delivery

**DOI:** 10.1002/advs.202308659

**Published:** 2024-01-28

**Authors:** Qiaoxia Zhou, Qiongliang Liu, Yan Wang, Jie Chen, Otmar Schmid, Markus Rehberg, Lin Yang

**Affiliations:** ^1^ Institute of Lung Health and Immunity (LHI), Helmholtz Munich Comprehensive Pneumology Center (CPC‐M) Member of the German Center for Lung Research (DZL) 85764 Munich Germany; ^2^ Department of Forensic Pathology West China School of Preclinical and Forensic Medicine Sichuan University No. 17 Third Renmin Road North Chengdu 610041 China; ^3^ Burning Rock Biotech Building 6, Phase 2, Standard Industrial Unit, No. 7 LuoXuan 4th Road, International Biotech Island Guangzhou 510300 China; ^4^ Department of Thoracic Surgery Shanghai General Hospital Shanghai Jiao Tong University School of Medicine Shanghai 200080 China; ^5^ Qingdao Central Hospital University of Health and Rehabilitation Sciences (Qingdao Central Medical Group) Qingdao 266042 China; ^6^ Department of Respiratory Medicine National Key Clinical Specialty Branch of National Clinical Research Center for Respiratory Disease Xiangya Hospital Central South University Changsha Hunan 410008 China; ^7^ Center of Respiratory Medicine Xiangya Hospital Central South University Changsha Hunan 410008 China; ^8^ Clinical Research Center for Respiratory Diseases in Hunan Province Changsha Hunan 410008 China; ^9^ Hunan Engineering Research Center for Intelligent Diagnosis and Treatment of Respiratory Disease Changsha Hunan 410008 China; ^10^ National Clinical Research Center for Geriatric Disorders Xiangya Hospital Changsha Hunan 410008 P. R. China

**Keywords:** advanced imaging, intracellular delivery, nano‐bio interaction, nano‐drug‐carries, physiological models

## Abstract

Intracellular delivery of nano‐drug‐carriers (NDC) to specific cells, diseased regions, or solid tumors has entered the era of precision medicine that requires systematic knowledge of nano‐biological interactions from multidisciplinary perspectives. To this end, this review first provides an overview of membrane‐disruption methods such as electroporation, sonoporation, photoporation, microfluidic delivery, and microinjection with the merits of high‐throughput and enhanced efficiency for in vitro NDC delivery. The impact of NDC characteristics including particle size, shape, charge, hydrophobicity, and elasticity on cellular uptake are elaborated and several types of NDC systems aiming for hierarchical targeting and delivery in vivo are reviewed. Emerging in vitro or ex vivo human/animal‐derived pathophysiological models are further explored and highly recommended for use in NDC studies since they might mimic in vivo delivery features and fill the translational gaps from animals to humans. The exploration of modern microscopy techniques for precise nanoparticle (NP) tracking at the cellular, organ, and organismal levels informs the tailored development of NDCs for in vivo application and clinical translation. Overall, the review integrates the latest insights into smart nanosystem engineering, physiological models, imaging‐based validation tools, all directed towards enhancing the precise and efficient intracellular delivery of NDCs.

## Graphical abstract

This comprehensive review explores recent advancements in intracellular NP delivery, emphasizing the design of personalized nano‐drug‐carriers for clinical applications. It highlights the importance of evaluating drug efficacy and delivery efficiency in physiologically relevant ex vivo human tissue models. It also underscores the significance of employing state‐of‐the‐art microscopic techniques to study nano‐bio interactions.

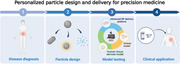

## 1 Introduction

Taking advantage of nanotechnology‐enabled disease prevention, monitoring, and therapy, scientific investigation into nanomedicine has undergone a remarkable outburst in the last decades.^[^ ^1^ ^]^ Nano‐drug‐carriers (NDC) have been developed to overcome the challenges faced by traditional free drugs and offered tremendous potency in various biomedical application scenarios, for example, nanoparticles (NPs) were used for imaging‐guided surgery, regional heat or radiation therapy to tumors,^[^ ^2^ ^]^ encapsulation of mRNA vaccines against COVID‐19,^[^ ^3^ ^]^ manufacture of miniaturized medical devices, and foremost drug delivery.^[^ ^4^ ^]^ NPs served as NDC could enhance the stability, solubility, and biocompatibility of encapsulated cargos by promoting the transporting ability across membranes, and prolonging circulation time which are the bottlenecks for conventional formulations. Due to the improved delivery capability of drugs to specific organs, tissues, and even cells leveraged by intelligent nanotechnology, NP research was exponentially outbreak, producing promising results e.g., high‐dose of NP accumulation in lesions and tumors, selective organ delivery, enhanced drug efficacy, and reduced side effects in in vitro and in vivo studies.^[^ ^5^ ^]^ NP formulations could further reduce the degradability of pharmaceutical molecules and implement precise drug release in a stimulus‐triggered or sustained manner.^[^ ^6^ ^]^ Combined drug delivery is generally not feasible via conventional free drug administration. NP delivery systems were thus designed or synthesized into complex architectures, allowing for multi‐parallel delivery of, e.g., bio‐responsive moieties, targeting agents, and therapeutics to inflamed or diseased regions which maximizes drug efficacy.^[^ ^7^ ^]^ Additionally, spatial visualization and real‐time monitoring of NDC in cells, organs, and organisms can be achieved by the integration of fluorophores and cargoes into NDC systems or using fluorescent NDC combined with advanced imaging technologies.^[^ ^8^ ^]^

Despite several NP formulations (i.e., Doxil and Abraxane) are currently being used in the clinic for cancer therapy, most of NDC are unavailable to patients which can be attributed to, e.g., overstated functions of NDC, the inefficiency of preclinical studies, translational gaps between animal and human studies, and disease heterogeneity and complexity.^[^ ^1^ ^]^ Among multiple challenges in NDC delivery, the poor efficiency of delivering agents to target sites or cells and obscure transporting mechanisms are the main obstacles hindering controlled and improved therapeutic efficacy. For instance, Chan group has previously demonstrated that NPs transportation occurs through the trans‐endothelial pathway rather than passive diffusion through endothelial gaps.^[^ ^9^ ^]^ They recently propose that NPs leaving tumors via lymphatics reenter the bloodstream, recirculating to enter the tumor again, which is termed as active transport and retention, challenging the conventional enhanced permeability and retention (EPR) theory.^[^ ^10^ ^]^ Variations in NP characteristics, cell types, and disease endotypes also contribute to diverse internalization pathways as well as inconsistent uptake efficiency. The interactions between NPs and physiological fluids such as protein corona formation (opsonins) by absorbing surrounding proteins on NP surface could enhance the cellular recognition and clearance of NPs by the mononuclear phagocyte system, leading to deficient delivery of the NPs to targeted tissue, undesired accumulation in off‐target tissues, and unwanted side effects.^[^ ^11^ ^]^

This review thus presents advances in highly efficient in vitro intracellular delivery, predominantly focusing on emerging platforms for passive intracellular NP delivery and briefly summarizing active endocytic routes that have been widely discussed somewhere else.^[^ ^12^ ^]^ Passive NP delivery is generally realized via membrane disruption‐enabled techniques, in which the external (electrical, thermal, optical, or mechanical) energy is applied to the cells triggering membrane deformation and permeability for the exogenous cargo. Depending on the external sources, this review highlights passive NP delivery strategies based on mature technology platforms like electroporation, sonoporation, photoporation, microfluidic method, and microinjection. Compared to active endocytosis executed by targeted and non‐targeted cells, those passive delivery methods theoretically possess several merits like high delivery efficiency, easy‐to‐control, and high throughput. This review further elaborates on the impact of NP physicochemical characteristics including NP size, shape, surface charge, hydrophobicity, and elasticity on cellular uptake and discusses several complex NDC systems aiming for hierarchical targeting and delivery in vivo for generation and engineering of precision NP in the future. In contrast to widely used single cell line for NP delivery, emerging physiological in vitro and ex vivo models derived from animal/human specimens could better mimic the in vivo delivery environment and potentially fill the gaps between species (i.e., mice versus humans) are also explored, which might be of particular importance as new avenues arise for future clinical translation of NDC in drug delivery and cancer nanomedicine. The discussion of high‐resolution and rapid‐evolving microscopical methodologies for NP imaging and tracking provides comprehensive insights into the distribution, localization, and interaction of NDC within the cell, organ, and organism, eventually prompting the application of nanotechnology in biomedicine. In summary, this review primarily emphasizes three core aspects of NP delivery science: NP delivery systems, physiological models, and methodological tools (imaging), tailored to the specific content requirements. It is important to note that due to the scope of the review, it may not comprehensively cover all NP types (e.g., metal, lipid, polymeric NPs). It underscores the importance of prioritizing human tissue‐derived models and evidence‐based strategies to enhance the design of effective nanosystems.

## 2 Cellular Uptake Pathways for Nano‐Drug‐Carriers (NDC)

**Figure 1:**
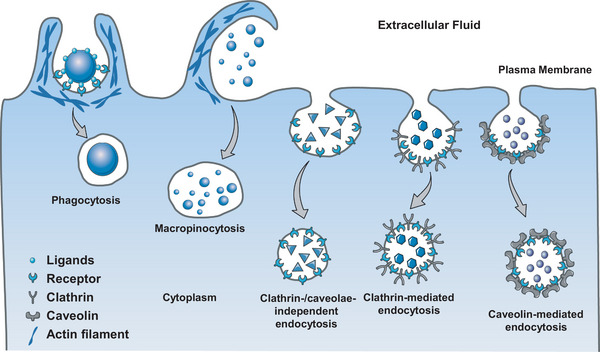
Schematic illustration of the main endocytic pathways for NPs entry into cells. Five pathways including multiple endocytosis and phagocytosis are predominantly involved in active NP cellular uptake.

Unraveling the molecular mechanisms of NP cellular uptake is of critical importance for the development of novel delivery strategies together with smart‐designed NDC facilitating highly efficient in vitro, ex vivo, and in vivo intracellular transportation.^[^ ^13^ ^]^ There is, however, still inadequate knowledge regarding the NP cellular uptake pathways due to, e.g., the complexity of the ingestion process, numerous molecules involved in multiple uptake pathways, the lack of specific pathway inhibitors, and heterogenous uptake behaviors for different cell types.^[^ ^14^ ^]^ It has been reported that NP internalization into mammalian cells is mainly through active endocytosis including phagocytosis and pinocytosis for large particles and fluids/solutes, respectively.^[^ ^12a^ ^]^ Phagocytosis is performed by multiple immune cells during innate immune responses caused by foreign NPs and microorganisms, while other non‐immune cells utilize pinocytosis pathways for intracellular uptake that can be subdivided into macropinocytosis, clathrin‐/caveolae‐mediated endocytosis, and clathrin‐/caveolae‐independent endocytosis^[^ ^1^, ^12^ ^]^ (**Figure** **1**). As a crucial mean of pathogen elimination, phagocytosis is mainly executed by specialized phagocytes, including macrophages/monocytes, neutrophils, eosinophils, and dendritic cells.^[^ ^15^ ^]^ Recognized by receptors on the cell membrane, NPs are swallowed by phagosomes and end up in fusion with lysosomes, which eventually forms phagolysosomes in the cytoplasm.^[^ ^12b^ ^]^ Improving the recognition of the particles by cells has been considered an effective way to enhance cellular uptake, and thus various strategies such as coating NPs with immunoglobulins,^[^ ^16^ ^]^ complement proteins, and other molecules have been developed. Besides, the size and shape of the particles and curvature of the interface between the particle and cell membrane at the initial contact also affect the efficiency of cellular internalization, for example, optimization of the morphological index of the particles has been applied to increase cell loading by phagocytosis.

Unlike phagocytosis, pinocytosis can be executed by almost every eukaryotic cell including endothelial, epithelial, and mesenchymal cells. Macropinocytosis is a unique pinocytosis process of nonspecific bulk fluid uptake which is not involved in the utilization of cargo.^[^ ^17^ ^]^ Cytoskeletal rearrangement was induced by growth factors, bacteria, viruses, necrotic cells, and NPs, resulting in membrane ruffling and extension, followed by engulfment of the cellular milieu. In clathrin‐ or caveolae‐mediated endocytosis (CME and CvME), either clathrin or dimeric protein caveolin‐1 is indispensable for the formation of the vehicles, which are responsible for cell signaling, cellular homeostasis, lipid regulation, and vesicular transport.^[^ ^18^ ^]^ There are various pathways involved in clathrin‐ and caveolae‐independent endocytosis, including the endocytosis process in CME/CvME‐depleted cells. These pathways are involved in delivering cellular fluids and molecular compounds such as interleukin‐2, glycosylphosphatidylinositol (GPI)‐linked proteins, growth hormones, and folic acid.^[^ ^19^ ^]^ Significant progress in understanding the clathrin‐independent pathway has been made through the discovery of key molecules and regulators including Cdc42, galectin‐3, endophilin, and IRSp53.^[^ ^20^ ^]^ The endocytic uptake of NPs has been sophisticated discussed in the recent literature,^[^ ^12^, ^15^ ^]^ this review thus focus on introducing, e.g., non‐endocytosis‐based intracellular delivery platforms, novel cell/organ models, smart in vivo NP delivery systems, and emerging NP‐tracing tools to provide systemic insights into cell delivery and guidance for future safe‐by‐design NDC.

## 3 Advanced Platforms for NDC Cellular Delivery

### 3.1 Electroporation‐Assisted NP Delivery

**Figure 2:**
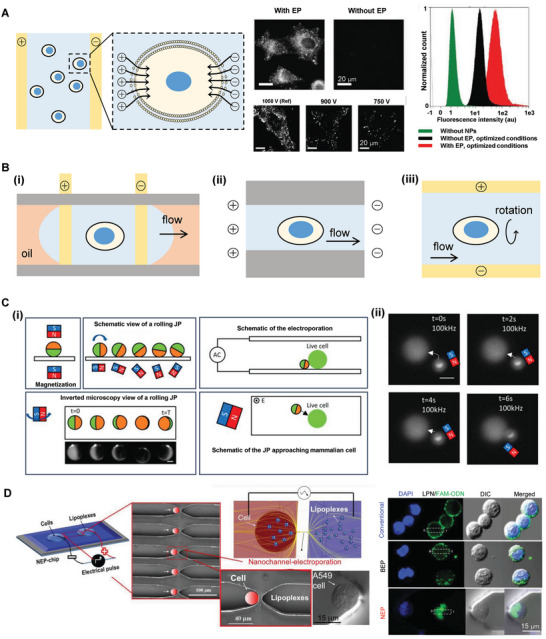
Electroporation strategies for intracellular delivery. A) Electroporation of suspended cell group with parallel plate cuvette. The orientation and polarization effect generate transient pores on the cell membrane in an electric field, with flow of positive charges into the cells after failure of the lipid bilayer (left). The electroporated Hela cells incubated with dye‐loaded methyl methacrylate‐based polymers (PMMA) NPs exhibited higher fluorescent intensity compared with those without electroporation (EP), and the uptake efficiency is associated with pulse intensities. Reprinted with permission,^[^ ^27^ ^]^ Copyright 2020, Wiley‐VCH. B) Single cell electroporation strategies with oil droplets (i), limited pipe diameter (ii), and cell rotation for homogeneous permeabilization (iii). C) Schematic illustration of a localized precise electroporation approach by magnetically and electrically powered metallodielectric hybrid micromotor (i). Sequential images displayed the pathway of a 5‐um JP particle approaching and contacting a K562 cell with the magnetic‐guided electroporation (ii, scale bar = 10 um). Reprint with permission.^[^ ^32^ ^]^ Copyright 2021, National Academy of Science. D) Schematic illustration and SEM images of a novel nanochannel‐based device, in which two microchannels connected by a nanochannel as a unit for the precise placement of individual cell and enables direct targeted transfection via voltage pulses (left). Confocal microscopy images demonstrated the direct injection of lipoplex NPs into cell cytoplasm via NEP, while the NPs were attached to the cytomembrane in conventional incubation and bulk electroporation (BEP) methods (right). Reprinted with permission.^[^ ^34^ ^]^ Copyright 2014, Wiley‐VCH.

Electroporation (EP) is known as the permeabilization of the cytomembrane by electric pulses, forming pores on the cell membrane as channels for transporting foreign substances into the intracellular environment, which was initially used as a (genetic) modification method through delivering DNA, proteins, and other biomacromolecules to mammalian cells.^[^ ^21^ ^]^ With the development of nanotechnology, electroporation was then utilized to meet the imperious demands of delivering NPs, which could modulate three key barriers affecting intracellular drug concentration including microvascular permeability,^[^ ^22^ ^]^ extracellular matrix,^[^ ^23^ ^]^ and cell membrane.^[^ ^24^ ^]^ Transient or permanent permeabilization can be achieved using either reversible electroporation (RE) or irreversible electroporation (IRE), respectively.^[^ ^21b^ ^]^ Access of NPs into cells requires temporary opening of channels and subsequent self‐repair of the cytomembranes for particle retention, therefore RE was thus most commonly used,^[^ ^25^ ^]^ while IRE could cause cell death through the breakdown of homeostasis, since it was developed for tumor ablation directly serving as a therapeutic method.^[^ ^26^ ^]^ Traditional electroporation strategies are mainly targeted at cell bulks with the orientation and polarization effect generating transient pores on the cell membrane in an electric field, followed by the flow of positive charges into the cells after the failure of the lipid bilayer, which results in efficient batch processing of cell permeabilization^[^ ^27^ ^]^ (**Figure** **2**A). Current approaches have explored the potential of microchannel‐based electroporation, which enables more homogeneous permeability and higher penetration efficiency for individual cells^[^ ^28^ ^]^ (Figure 2B). Under precisely controlled electroporation, only the constructed cells can undergo irreversible damage, leading to various cell death pathways, including apoptosis, necrosis, and immunogenic cell death like necroptosis and pyroptosis, while adjacent cells, tissues, and scaffolds remain intact, preserving normal physiological functions.^[^ ^29^ ^]^ This provides the foundation for the clinical application of electroporation‐mediated NP delivery.

Parameters impacting the effect of EP can be determined by multiple factors like the targeted molecules, the voltage of pulses, exposure time, frequency, and the number of electric shocks.^[^ ^21a^ ^]^ Long‐lasting electrical pulses are required for the internalization of nucleic acids into the cytoplasm or nucleus, while transient permeabilization within the cytomembrane is enough for small hydrophilic molecules. Derek et al. reported the enhanced intracellular uptake of iron oxide NPs by PANC‐1 cells in an in vivo model of pancreatic ductal adenocarcinoma, which exhibited a significant time‐dependent manner according to the duration of electroporation application.^[^ ^30^ ^]^ Jeong et al. investigated the direction of silica NP‐gene complex electroporative internalization using microscale electroporation, in which the anionic NPs displayed more significant enhanced uptake compared with the cationic ones, exhibiting greater potential as carrier in the development of electroporation‐based gene/drug delivery platforms.^[^ ^31^ ^]^ A magnetically and electrically powered metallodielectric hybrid micromotor was developed, which enabled control of the particle direction and movement approaching targeted cells through magnetic steering. With the limited cell membrane contacting the motor, this strategy achieved localized, partially precise electroporation^[^ ^32^ ^]^ (Figure 2C). Chen et al. explored the long‐term behavior of polystyrene core NPs as the “hard‐uptake” NPs after the electroporation and found that the NPs with smaller size exhibited better uptake efficiency as a transient response to electroporation, while the larger ones precipitated on the cytomembranes due to gravity and delivered fewer genes to the cells.^[^ ^33^ ^]^ Boukany et al. demonstrated that lipoplex NPs can be pushed into the cytoplasm directly under a nanochannel electroporation device within several seconds, and further proved that the siRNA assembled on lipoplex NPs has been without prior internalization of NPs by the endocytosis route, which suggested the great potential of lipoplex NPs in combination with electroporation for delivering large plasmids^[^ ^34^ ^]^ (Figure 2D). Besides, additional improvement in electroporation for NP delivery has been proposed, for example, Jen et al. demonstrated that electromigration following electroporation significantly enhanced the transportation efficiency of gold NPs (AuNPs) compared to the electroporation alone, which could be explained by the additional support by the increased concentration of NPs on the cell membrane after electrophoretic migration.^[^ ^35^ ^]^ In animal models, electroporation enhances the delivery of sorafenib NPs^[^ ^36^ ^]^ and radiolabeled liposomal doxorubicin NPs (89Zr‐NRep)^[^ ^37^ ^]^ to tumors by directly affecting transfection and modulating vascular permeability and the extracellular space.

### 3.2 Sonoporation‐Assisted NP Delivery

**Figure 3:**
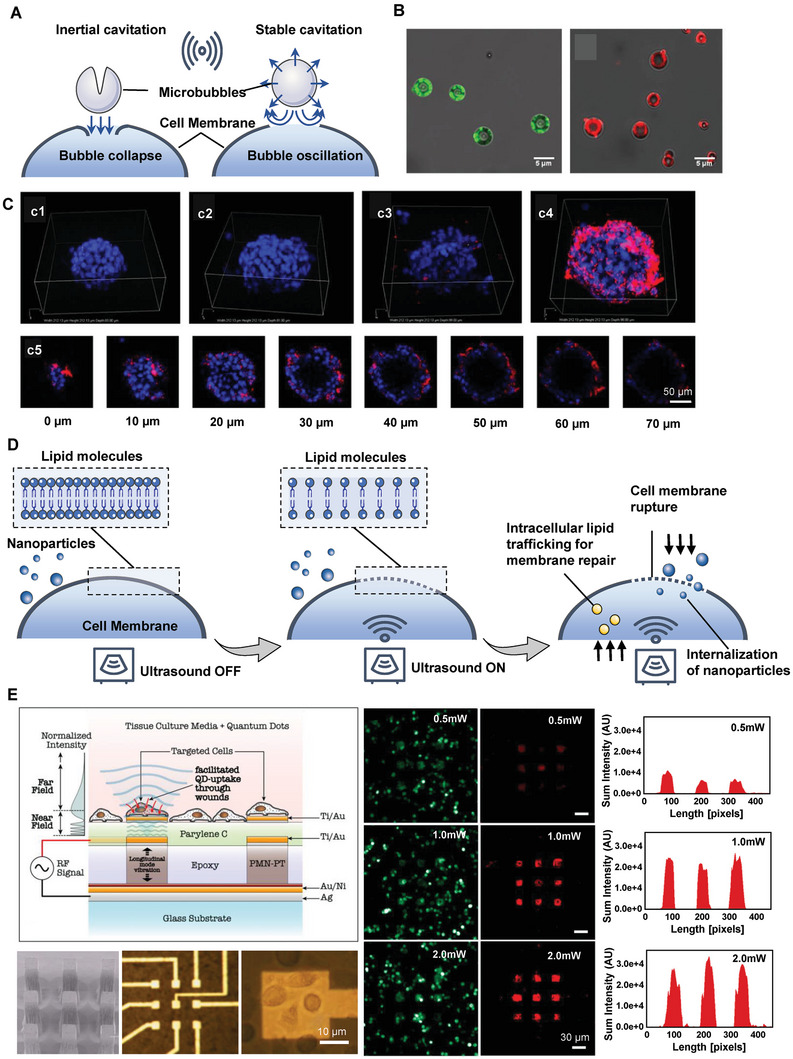
Sonoporation strategies for intracellular delivery. A) Schematic illustration of shear force generation with microbubble‐based ultrasound via the collapse (left) or oscillation (right) of a cavitation bubble. B) Representative confocal imaging of microbubbles loaded with 100 nm fluorescent polystyrene beads (green) and DiD‐labeled liposomes (red). C) Sonoprinting of NP microbubbles on 4T1 tumor monospheroids. Confocal Z‐stack maximum intensity projections from: c1) an untreated spheroid, c2) a spheroid exposed to DiD‐labeled liposomes alone, c3) a spheroid exposed to coadministered DiD‐labeled liposomes and microbubbles with ultrasound, and c4) a spheroid exposed to DiD‐labeled liposomes coupled onto microbubbles with ultrasound (sonoprinting). White box dimensions: 212 µm x 212 µm x 90 µm. C5: Sequential cross sections of the spheroid in c4) at increasing depth. Reprinted with permission,^[^ ^52^ ^]^ Copyright 2019, Elsevier B.V. D) Schematic illustration of cell membrane permeabilization via sonoporation. E) Schematic illustration of a novel site‐specific sonoporation device, showing the cells seeded on the ultrasonic micro‐transducer arrays (UMTA) and treated with RF signal‐induced ultrasonic waves through the PMN–PT transducer (left), and the fluorescence images showed the quantum dots (red) uptake by LU1205 cells seeded on the array (green) in a RF power‐dependent manner (right). Reprinted with permission,^[^ ^61^ ^]^ Copyright 2011, Elsevier B.V.

As a non‐invasive and cost‐effective option, ultrasound has not only been extensively applied in preclinical research such as molecular and organ imaging but also is frequently used in clinics for disease diagnosis and therapeutics.^[^ ^38^ ^]^ Cell membranes were disrupted by the acoustic waves generated from ultrasound for enhanced intracellular delivery of both NPs and biomacromolecules which is termed sonoporation. However, the sole application of high‐intensity ultrasound possesses poor enhancement of NPs permeation into tissues. Ultrasound‐mediated drug delivery can thus be generally amplified by using commercial microbubbles.^[^ ^39^ ^]^ The fundamental principles of ultrasound‐mediated NPs internalization mainly include acoustic radiation force, tissue heating, and cavitation, followed by the biophysical effects on the nearby cell surface including the push‐pull effect by bubble vibration, shear stress by microstreaming, and jet streams or shock waves by bubble collapse,^[^ ^38^, ^40^ ^]^ all of which result in sonoporation and enhance the permeability of tissue and cells, as well as the penetration and accumulation of the NPs (**Figure** **3**A).

Compared with endogenous microbubbles, exogenous ones exhibited a lower threshold of cavitation effect, and a variety of commercial microbubbles have been assessed in vitro experiments, such as Definity,^[^ ^41^ ^]^ Lumason,^[^ ^42^ ^]^ Optison,^[^ ^43^ ^]^ Sonazoid,^[^ ^44^ ^]^ Sonovue,^[^ ^45^ ^]^ and Targestar^[^ ^46^ ^]^ with diameters ranging from 1 to 4 µm. Furthermore, small molecules were applied to sonoporation‐mediated delivery including propidium iodide,^[^ ^47^ ^]^ dextrans,^[^ ^48^ ^]^ and calcein^[^ ^49^ ^]^ with sizes ranging from several to tens of nanometers. In addition, several studies have developed custom‐made microbubbles loaded with drugs for efficient delivery of therapeutic agents.^[^ ^50^ ^]^ For drugs prone to rapid metabolism, degradation, or non‐specific toxicity to organs, loading them onto NPs for microbubble‐mediated internalization or encapsulating them within microbubbles could offer effective delivery options to diseased or tumor sites. This approach enhances pharmacodynamics and pharmacokinetics while reducing side effects. For instance, during ultrasound radiation, NP‐loaded microbubbles exhibited a much higher cell membrane deposition and cytosolic internalization compared to the simple mixture of NPs and microbubbles in vitro.^[^ ^51^ ^]^ This processed was termed as “sonoprinting”. The group further investigated whether sonoprinting can be achieved in physiologically relevant 3D tumor spheroids. They indeed showed that sonoprinting enables to deliver substantial quantities of doxorubicin‐containing liposomes onto the outer cell layers of spheroids and release of doxorubicin into the deeper layers of the spheroids, resulting in a marked reduction in cell viability^[^ ^52^ ^]^ (Figure 3B,C). Elevated acoustic pressures (>300 kPa) and longer ultrasound pulses (>100 cycles) are crucial for efficient sonoprinting, where released NPs are transported alongside microbubbles to deposit on the cell membrane.^[^ ^53^ ^]^

To achieve both efficient delivery and minimal damage to normal cells and tissues, several crucial parameters of ultrasound have to be selected properly. The ultrasound frequency covers a wide range from hundreds of kHz to a few MHz, which is a relatively lower frequency to reach deeper tissues by avoiding increased acoustic attenuation when compared with that for diagnostic applications.^[^ ^54^ ^]^ And a resonance frequency should be calculated utilizing the parameters of the surrounding medium and composition/size of the microbubbles, which provide reference for selecting the optimum insonation frequency in stable cavitation. Besides, the intensity of ultrasound should always be kept under the safety limits (0.3–3 W cm^−2^) in combination with appropriate duty cycles to achieve a suitable temporal‐average ultrasound intensity and avoid irreversible thermal damage to normal cells.^[^ ^55^ ^]^ In addition, long enough ultrasound exposure time is essential for the destruction of microbubbles, which was determined by pressure amplitude.^[^ ^40^ ^]^ Thus, high‐pressure amplitudes are generally consumed for inertial cavitation, which allows more effectively destroyed microbubbles under shorter exposure time, achieving desired therapeutic outcome and reduced injury to healthy tissues.

In recent years, several studies have provided evidence for the effective delivery of NPs using different materials. A recently developed ultrasound‐responsive catalytic microbubbles loaded with piperacillin and Fe_3_O_4_ NPs to combat *P. aeruginosa* biofilm‐induced lung infections. These microbubbles disrupt biofilm structures, enhance drug penetration, chemically degrade biofilm matrices, and activate immune responses, effectively treating chronic lung infections in a mouse model, offering a promising strategy against bacterial biofilm infections.^[^ ^56^ ^]^ Lu et al. reported an increased intracellular concentration of AuNPs encapsulated in microbubbles and exposed to ultrasound, showing a dose‐dependent manner associated with the degree of inertial cavitation.^[^ ^57^ ^]^ Doyeon et al. modified the transarterial chemoembolization (TACE) method by developing a doxorubicin‐loaded NP conjugated microbubble as a new formulation, which exhibited an enhanced delivery of the NPs into liver tumors under ultrasound irradiation, and also improved the therapeutic efficacy of TACE against liver cancer.^[^ ^58^ ^]^ Maryam et al. successfully delivered poly‐ethyleneimine (PEI)‐coated mesoporous silica NPs (MSNs) into suspended tobacco cells using ultrasound treatment, but also discovered an adverse effect of high‐level sonoporation on cell viability,^[^ ^59^ ^]^ which inspired further studies into the safety of NPs delivery mediated by ultrasound.

Bubble‐based sonoporation relies on the dynamic behavior of microbubbles and necessitates additional contrast agents, which can compromise the approach's stability and add extra cost and complexity to the procedure.^[^ ^54^, ^60^ ^]^ Novel non‐bubble‐based sonoporation approaches have thus been developed, aiming to establish pores on the cell membrane by increased stress from either radiation force or streaming‐induced shear force of ultrasound, which could be utilized as a temporary channel for intracellular delivery^[^ ^38^ ^]^ (Figure 3D). In addition to the suspension cells, the adherent cultured cell can also accept forces by penetrative propagation of acoustic waves through the substrate, which in turn results in pores by additional membrane stress. For example, a transducer‐generated localized sonoporation system by utilizing piezoelectric micropillars arrays was developed for enhanced particle delivery.^[^ ^61^ ^]^ Magnesium niobate–lead titanate (PMN‐PT) transducers were connected to microelectrodes, which could be activated to produce ultrasound pressure to cells seeded on the biochip, and thus achieve cellular‐level enhanced delivery of quantum dots (QDs) to specified cells^[^ ^61^ ^]^ (Figure 3E). Besides, given the advantages of non‐bubble‐based strategies with less cell damage and higher cell viability, other bulk‐/surface‐based acoustic waves with different frequencies were developed for intracellular delivery of biomacromolecules, however, it is of great need to verify its utility in NP delivery.

### 3.3 Photoporation‐Assisted NP Delivery

**Figure 4:**
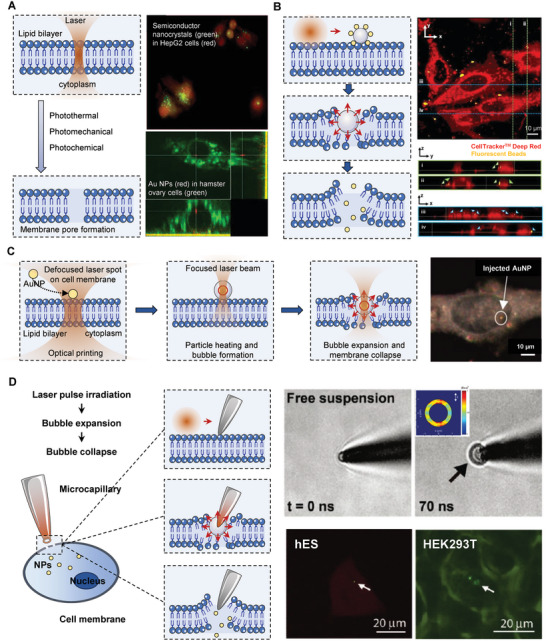
Photoporation strategies for intracellular delivery. A) Laser photoporation directly on cell membrane with energy absorbed by the plasma membrane. For example, the semiconductor nanocrystals photoinjected into human hepatocyte HepG2 cells. Reprinted with permission,^[^ ^64^ ^]^ Copyright 2006, SPIE. And AuNPs (red) delivered into the nucleus of a hamster ovary cell (green) by laser photoporation, with visualization of confocal cross sections in xy (main), xz (bottom), and yz (right). Reprinted with permission,^[^ ^63^ ^]^ Copyright 2009, Wiley‐VCH. B) The water vapor nanobubble‐based photoporation. A light‐triggered self‐assembled nanobombs (NBs), which consist of a photothermal core particle surrounded by smaller nanoprojectiles. The core particle is photoconductive and could be heated by irradiation of pulsed laser light, which results in vapor bubble formation. The expansion of the vapor bubble propels the nanoprojectiles through the cell membrane of nearby cells (left), which achieves efficient penetration of nanoprojectiles (indicated with narrow heads) into the cell plasm (red). Reprint with permission,^[^ ^68^ ^]^ Copyright 2022, Springer Nature. C) A two‐step variable laser for delivering gold NPs. Suspended particles were firstly printed on cell membrane by a defocused laser spot, and then the captured particles were heated by focused laser beam, which created vapor nanobubbles around particles, and consequently directly pushed into cells bubble expansion. As a result, thish plasmonic heating and optical force‐based platform has achieved effective delivery of gold NPs with a cell viability of 70%. Reprinted with permission,^[^ ^69^ ^]^ Copyright 2015, American Chemical Society. D) A phtoporation based microinjector. With a Ti‐coated microcapillary, the injector can be heated with laser irradiation, which creates nanobubbles at the tip of syringe for membrane pore formation, allowing delivery of NPs (left). Thermal energy conducting away from the Ti film heats the adjacent thin water layer to above the critical temperature, generating a vapor nanobubble on the ring‐shaped micropipet tip (upper right), which delivered 100 nm and 200 nm green fluorescence beads into hES and HEK293T cells, respectively (lower right). Reprinted with permission,^[^ ^70b^ ^]^ Copyright 2011, American Chemical Society.

Photoporation, known as the technique utilizing optical energy to destroy the cytomembrane integrity and improve membrane permeability, has been developed as a promising strategy for intracellular delivery, which stands out from other approaches with multiple unique advantages. Typically, the laser beam is precisely illuminated on a small spot (1–10 µm) of the cell membrane with a short pulse duration (femtoseconds to nanoseconds) to generate intensive optical energy. Based on various possible mechanisms, such as photothermal, photomechanical, and photochemical procedures, pores are created on the membrane of specific cells, allowing internalized diffusion of exogenous NPs.^[^ ^62^ ^]^ For instance, using direct laser‐induced photoporation, targeted injection of AuNPs^[^ ^63^ ^]^ and semiconductor nanocrystals^[^ ^64^ ^]^ into mammalian cells have been applied, suggesting photoporation as a novel alternative for precision‐targeted delivery in a single cell (**Figure** **4**A). However, the disadvantages of photoporation include the high cost of laser devices and time‐consuming procedure in batch delivery.

To address these challenges, nanomaterials were added to photoporation as sensitizers including both single nanostructures (e.g., AuNPs, iron oxide nanofibers, graphene quantum dots, and polydopamine NPs)^[^ ^65^ ^]^ and composite nanostructures (polymeric films and nanofibers containing photothermal NPs as well as composite nanoscale biolistic nanostructures).^[^ ^62^, ^66^ ^]^ For metallic NPs, the free electrons inside particles will oscillate with the greatest amplitude when the input laser frequency matches the resonant frequency of the localized surface plasmons, which is a phenomenon known as localized surface plasmon resonance (LSPR).^[^ ^67^ ^]^ Subsequently, a series of sequential energy transfer processes occur, leading to the thermalization of NPs, and the heated NPs can cause thermal denaturation of integral glycoproteins, which results in cell membrane perforation at the contact area with the sensitizer NPs. This thermalization process can be achieved by continuous wavelength laser light, which is relatively less expensive, however, it requires up to a few minutes for pore formation limiting the throughput.

The NP temperature can be extremely high when employing powerful and transient laser beams (<100 ps) due to the failed diffusion of heat into the environment. As a result, the temperature of the NP can rapidly rise to even thousands of degrees, causing the surrounding water to evaporate and create water vapor nanobubbles.^[^ ^65b^ ^]^ With strong shockwaves and liquid jets, bubbles easily expand and collapse, creating channels for intracellular delivery. For instance, Fraire et al. have introduced light‐triggered self‐assembled nanobombs (NBs), which consist of a photothermal core particle surrounded by smaller nanoprojectiles. The core particle is photoconductive and could be heated by irradiation of pulsed laser light, which results in vapor bubble formation. The expansion of the vapor bubble can propel the nanoprojectiles through the cell membrane of nearby cells, which achieves efficient penetration of nanoprojectiles into the cytoplasm. They outperform electroporation by 5.5‐7.6 times in transfection yield, offering high‐throughput and scalable mammalian cell transfection^[^ ^68^ ^]^ (Figure 4B). In addition to the metallic sensitizers, carbon‐based nanostructures can also serve as intermediates for bubble generation. The carbon‐steam reaction can create carbon monoxide and hydrogen gas, which induces cavitation shockwaves.^[^ ^66^ ^]^ However, most studies utilizing carbon nanomaterials have only explored its application in the transfection of macromolecules, the potential feasibility for cytoplasmic transferring of other NPs is to be explored.

Optimized photoporation strategies based on standard modes have also been developed. Li et al. have introduced a two‐step variable laser for delivering AuNPs^[^ ^69^ ^]^ (Figure 4C). Suspended particles were first printed on the cell membrane by a defocused laser spot, and then the captured particles were heated by a focused laser beam, which created vapor nanobubbles around particles. Consequently, the expansion and collapse of bubble lead to membrane rupture and the formation of a transient hole. The NPs are then immediately pushed into the cell by the optical force. As a result, this plasmonic heating and optical force‐based platform has achieved effective delivery of AuNPs with cell viability of 70%.^[^ ^69^ ^]^ Inspired by the sensitizer‐assisted photoporation, Chiou's lab has developed a novel nanoblade for delivery.^[^ ^70^ ^]^ With a Ti‐coated microcapillary, the injector can be heated with laser irradiation, which creates nanobubbles at the tip of the syringe for membrane pore formation, allowing delivery of NPs, as well as untransferable large cargos into live cells beyond size limitations^[^ ^70b^ ^]^ (Figure 4D).

### 3.4 Microfluid‐Based NP Delivery Platform

**Figure 5:**
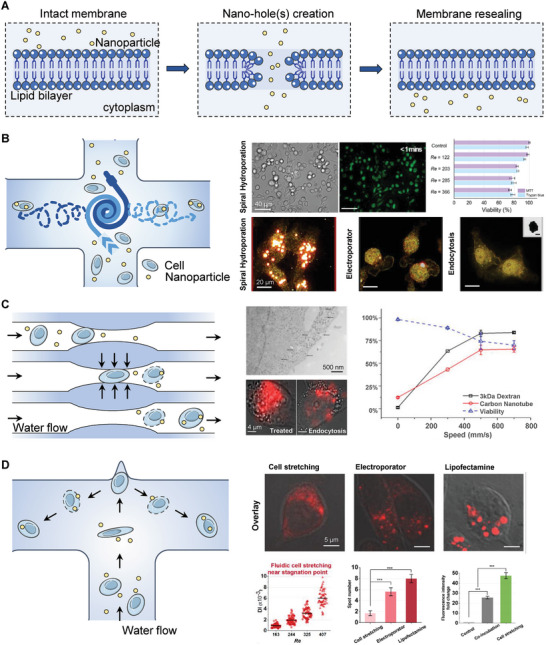
Microfluid‐based delivery platforms for intracellular delivery. A) Schematic illustration of the mechanical hole‐generation, membrane permeabilization, and resealing during the mechanical squeezing of the cells. Transportation of NPs are achieved instantaneously through the crack in the lipid bilayers. B) A spiral hydroporation platform developed with a cross junction and two water flows for intracellular delivery of NPs. Water flows mixed with cells and NPs were pumped with different velocity oppositely, and then exit in opposite directions, generating strong spiral vortex for deformation of cell membrane at the cross junction. Compared with electroporation and endocytosis delivery, the spiral hydroporation device achieved effective intracellular delivery of NPs and guarantees cell viability with moderate Reynolds numbers. Reprinted with permission,^[^ ^76^ ^]^ Copyright 2020, American Chemical Society. C) A silicon slice etched with sealed parallel microfluidic channels for intracellular delivery of NPs. Cells and NPs were driven by gas pressures through constrictions with width of 4–8 µm and length of 10–40 µm. Compared with endocytosis, the microfluidic channels achieved more effective intracellular delivery of carbon nanotubes. Reprinted with permission,^[^ ^77^ ^]^ Copyright 2013, National Academy of Science. D) A T‐junction channel with a cavity as a novel platform for NPs intracellular delivery. In virtue of the intrinsic inertial flow, an elongational recirculating flows was developed in the channel, leading to substantial cell stretching and discontinuity of lipid bilayer, which allows internalization of NPs. This new approach has been provided to delivery quantum dots and large particles with diameters of 300 nm, with high efficiency superior to conventional electroporation and lipofectamine methods. Reprinted with permission,^[^ ^78^ ^]^ Copyright 2020, American Chemical Society.

Similar to sonoporation and laser‐induced cavitation, the initial idea of the microfluid‐aided delivery was based on the fluid shear forces by fast water flow, which can induce cell deformation and disrupt lipid bilayers for the construction of intracellular delivery channels.^[^ ^71^ ^]^ With membrane resealing after the temporary deformation, this method is of high interest for intracellular delivery due to the minimal effect on cell viability^[^ ^28b^ ^]^ (**Figure** **5**A). To generate shear force with high intensity, the simplest method is the transflux of liquid through cramped constrictions. For instance, as early as the year 1992, McNeil et al. introduced syringe loading as an intracellular delivery approach. Cell suspension mixed with cargos was repeatedly aspirated and expelled through syringe needles, which produced shear forces around the entrance and exit of the needle and thus promoted cell permeabilization and intracellular delivery.^[^ ^72^ ^]^ Subsequent in vitro studies have revealed the utilization of syringe‐based approaches in delivery applications of plasmid, lightweight nucleotides, and proteins.^[^ ^73^ ^]^ Identically, another microfluidic device equipped with parallel constrictions was developed for delivering dextrans.^[^ ^74^ ^]^ However, the effectiveness of these methods for NP delivery is yet to be investigated.

To improve the intensity of fluid‐based shear force, Kizer et al. proposed a novel device, which generates fast water flow suspended with target cells and anticipant materials.^[^ ^75^ ^]^ Impaction between two opposite flows drives distributed cells into the cross‐junction center, inflicting strong shear stress on cells and inducing membrane deformation, which results in efficient and rapid delivery of large molecules (DNA and dextran) with high throughput. However, effective cytosolic delivery of NPs can only be achieved for small particles (less than 50 nm), which has limited further application of this platform in larger cargos.^[^ ^75^ ^]^ Thus, a modified spiral hydroporation platform was developed by Kang et al. to address this challenge.^[^ ^76^ ^]^ Cells were mixed with nanomaterials and pumped by two water flows with different velocities oppositely, and then exited in opposite directions (Figure 5B). The device with the design of cross junction introduces instability to the flows at the stagnation point where they meet and induces a strong spiral vortex for the deformation of the cell membrane, which enables robust and effective intracellular delivery of NPs and guarantees cell viability with moderate Reynolds numbers.

In addition to the spiral hydroporation‐based independent devices, other platforms tend to combine water flow with customized flow channels, which might induce cellular deformation or squeezing by constrictions. For example, a silicon slice etched with sealed parallel microfluidic channels was constructed as a novel device for intracellular delivery^[^ ^77^ ^]^ (Figure 5C). Cells were driven together with NPs by gas pressures through constrictions with a width of 4–8 µm and length of 10–40 µm, which enabled the effective delivery of QDs and carbon nanotubes into several cell types. Besides, another platform has designed a T‐junction channel with a cavity. In virtue of the intrinsic inertial flow, elongational recirculating flows were developed in the channel, leading to substantial cell stretching and discontinuity of lipid bilayer, which allows the internalization of NPs^[^ ^78^ ^]^ (Figure 5D). This new approach has been provided to deliver QDs and large particles with diameters of 300 nm into cells with high efficiency superior to conventional electroporation and lipofectamine methods.

Given the simplicity of the liquid‐based approaches which is independent of external power and intricate operation sequences, the disadvantages are equally apparent. Off‐target damages stand first the weakness, which may introduce injuries to the cytoskeleton, nucleus, and other organelles.^[^ ^79^ ^]^ To avoid unnecessary cell injury and achieve the balance between cell viability and effective delivery, optimal flow rates and modified compositions of the buffer are required. Researchers have indicated that the calcium supplementary in the flow buffer can promote rapid repair of membrane wounds, while a non‐calcium environment may maintain the membrane discontinuity for several minutes.^[^ ^80^ ^]^ In addition to the potential damages, the fixed constrictions have also limited the application of hydraulic power‐based platforms to specific cell types with similar sizes.^[^ ^28b^ ^]^ However, despite the drawbacks of the liquid‐based approaches, researchers have addressed the possibility of selective delivery to specific cell types in the heterogeneous mixture based on the cell size‐dependent platforms, which provided novel insights for targeted labeling of abnormally large cells (e.g., circulating tumor cells) for disease diagnosis.

### 3.5 NP Microinjection

**Figure 6:**
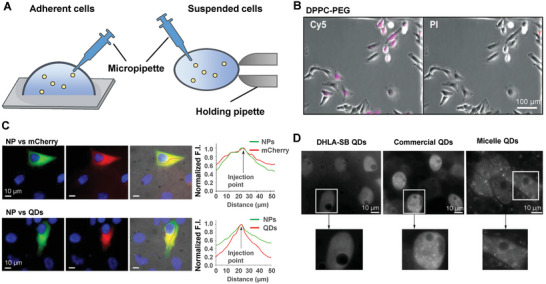
Intracellular delivery via microinjection. A) Schematic illustration of the microinjection operation of an adherent cell on a glass slide with a micropipette, or a suspended cell held by a secondary holding pipette. B) Microscopy images of human epithelial adenocarcinoma HeLa cells 4 h after injection of dipalmitoylphosphatidylcholine‐poly(ethylene glycol) (DPPC‐PEG), with injected cells shown in magenta (Cy5) and dead propidium iodide‐positive cells in red (PI). Reprinted with permission,^[^ ^85^ ^]^ Copyright 2017, American Chemical Society. C) Representative images of co‐injection in Hela cells (green) of NPs with mCherry (red, upper) or with quantum dots (QD, red, lower), together with the corresponding fluorescence intensity (right). Reprinted with permission,^[^ ^86^ ^]^ Copyright 2020, American Chemical Society. D) Distribution of microinjected NPs with three different surface chemistries. Under the microinjection circumstances, QDs coated with dihydrolipoic acid‐sulfobetaine (DHLA‐SB) ligands diffuse better in all cytoplasmic compartments suggesting the contribution of physicochemical properties in the intracellular fate of particles. Reprinted with permission,^[^ ^87^ ^]^ Copyright 2012, Wiley‐VCH.

Despite the efficiency of particle delivery, triggering the uptake of particles through endocytosis is not suitable for those with characteristics of prone degradation. To achieve precise compartment delivery inside the cells and avoid the inactivation of the loaded drugs due to the harsh environment of low pH and degradative proteases, microinjection is the most direct way to deliver particles into living cells.^[^ ^81^ ^]^ Using a nanoscale glass needle, the NPs as well as the loaded peptides, proteins, DNAs, and drugs could be physically penetrated to the cytomembranes.^[^ ^82^ ^]^ To implement the delicate operation, the adherent cells generally underwent the injection with supports such as coverslips and Petri dishes, otherwise, additional devices are needed for holding the cells suspended in supernatant, which will introduce redundant operations^[^ ^83^ ^]^ (**Figure** **6**A). The efficiency of NP internalization was compared between methods of microinjection and conventional incubation, and QDs were proved to remain in the cytoplasm for more than 24 hours after injection, while incubation‐mediated endocytosis particles were trapped in endosomal vesicles and did not penetrate the membrane.^[^ ^84^ ^]^ In addition to improved uptake efficiency, microinjection also preserved the surface properties of NPs, as well as avoided the endolysosomal barriers. Compared with conventional endocytosis by incubation with particle suspensions, microinjection tends to apply uniform distribution of particles instead of scrambled aggregation^[^ ^85^ ^]^ (Figure 6B). Theoretically, microinjection is a physical delivery method with the only variable of injected substance, and thus microinjection allows the accurate control of time, sites, and quantity^[^ ^86^ ^]^ (Figure 6C). However, studies have also demonstrated different intracellular distributions of microinjected particles with distinct sizes^[^ ^84^ ^]^ and surface chemistries^[^ ^87^ ^]^ (Figure 6D), suggesting the contribution of physicochemical properties to the intracellular fate of particles. Besides, based on the specific nucleus or mitochondrial localization sequence attached to QDs which could be preserved by microinjection, precise cellular sub‐localization and specific staining of cytoplasm or organelles are achievable.^[^ ^88^ ^]^ The labeled multiplexed QDs in combination with microinjection, which enables the tracking of single‐particle in the cytosol and precise directed targeting of NPs,^[^ ^89^ ^]^ and thus has been applied to fluorescence imaging techniques. In a recent study performed by Lee et al., microinjection was attempted in combination with flexible bioelectronic techniques for delivering light‐responsive theranostic NPs.^[^ ^90^ ^]^ Briefly, the isotonic NPs solution was contained in bioresorbable microneedles for targeted injection into tumors and located by magnetic resonance imaging, and then the particles were activated by high‐energy photons for accelerated diffusion of drugs. This technique is promising to overcome the blood‐brain barrier and achieve accurate delivery of NPs to targeted tissues.

### 3.6 Smart NP Delivery Systems with Functionalized Surface

Despite great scientific efforts have been invested in enhancing the efficiency of transcellular transport by modifying the characteristics of NPs, many of the above‐mentioned methods rely on specific cell types and material compositions, for example, the delivery approaches might be only worked out in exceptional conditions. Customized particles for specific cells have provided solutions for enhanced delivery efficiency in many cases but do not apply to transformed physical properties of particles. One of the flexible strategies for enhancing the active uptake of NPs by cells is to exploit the NP‐cell interactions, and thus smart NP delivery systems are gradually developed, for example, NPs modified with functional surfaces or specific ligands have been developed to improve the delivery efficiency, especially in the targeting of tumor cells or solid tumors in murine models. Unlike the alterations of morphology or physicochemical properties of NPs, surface functionalization or ligands encapsulation of NP systems is aimed at the enhanced cellular recognition and active uptake of the NPs. Among the functionalized surfaces, PEG,^[^ ^91^ ^]^ dextran,^[^ ^92^ ^]^ and chitosan^[^ ^93^ ^]^ were the most commonly used and the most effective molecules to improve biocompatibility of the NPs, evidenced by in vitro experiments. When it comes to improving active cellular uptake, intensive efforts have been made to developed novel surface‐modified particles by covalent binding of various molecules, including monoclonal antibodies or antibody antigen‐binding fragments (Fabs), natural proteins or small peptides, carbohydrates, aptamers, and other small molecules^[^ ^94^ ^]^ (**Table** **1**).

**Table 1:** Designing strategies of NPs surface ligands for enhanced active uptake.

Surface ligands of NPs	Targets	NPs	Study design	Cancer type	Cell line	Reference
Monoclonal Antibodies						
anti‐HER2 mAb	HER2	PLGA	in vitro	breast cancer	MDA‐MB‐453/MCF‐7/BT‐20	[313]
anti‐HER2 antibody	HER2	Golden NPs	in vitro in vivo	gastric cancer	N87	[314]
mAb against EGFR (Cetuximab)	EGFR	PLGA	in vivo	NA	NA	[315]
anti‐HSPG2 mAb	HSPG2/Perlecan protein	PLGA	in vitro	triple negative breast cancer	MDA‐MB‐231‐LM2	[316]
Antibody antigenbinding fragments (Fabs)						
anti‐HER2 Fabs	HER2	PEG‐PLGA	in vitro	breast cancer	SKBR3/MCF‐7/MDAMB‐436	[317]
anti‐EGFR Fabs	EGFR	PEG‐PLGA	in vitro	breast cancer	SKBR3/MCF‐7/MDAMB‐436	[317]
Natural proteins						
human serum albumin	SPARC	hybrid melanin‐silica‐silver NPs	in vitro	breast cancer	HS578T/MCF10a	[318]
transferrin	Tf‐cell receptor	doxorubicin‐loaded PLGA	in vitro	cervical cancer	HeLa	[319]
Small peptides						
K237	KDR/Flk‐1 protein	hybrid chitosan/poly(N‐isopropylacrylamide) NPs	in vitro	breast cancer	MDA‐MB‐231/L929	[320]
GE11	EGFR	nanomicelle containing evodiamine	in vivo	NA	NA	[321]
idiotype‐specific peptide	B‐cell receptor	diatomite‐based NPs	in vitro	myeloma	NA	[322]
Carbohydrates						
hyaluronic acid	CD44	carbon dots	in vitro	NA	4T1	[323]
mannose	Mannose receptor	PLGA‐PEG NPs	in vitro	NA (macrophage)	Raw 264.7	[324]
galactose	Galactose receptor	PLGA‐PEG NPs	in vitro	NA (macrophage)	Raw 264.7	[324]
dextran	Dextran receptor	PLGA‐PEG NPs	in vitro	NA (macrophage)	Raw 264.7	[324]
Aptamers						
CD133‐specific aptamer	CD133 receptor	lipid NPs	in vitro	osteosarcoma		[325]
Sgc8 aptamer	PTK‐7	mesoporous silica NPs	in vitro	acute T lymphocyte leukemia	CCRF‐CEM/Ramos	[326]
aptamer S‐MUC‐1	MUC1 protein	protein NPs	in vitro	breast cancer	MCF‐7	[327]
Small molecules						
folate	Folate receptor	chitosan‐lipid hybrid NPs	in vitro	ovarian cancer	SK‐OV‐3	[328]
anisamide	Sigma receptor	surface polymeric NPs	in vitro	colon cancer	HT‐29/HCT‐116/Caco‐2	[329]
phenylboronic acid	Sialic Acid	soy protein‐based NPs	in vitro	liver cancer	HepG2/SH‐SY5Y	[330]

In addition, altering NP surface components for immune escape has been proposed as an alternative approach to diminish clearance by the mononuclear phagocyte system, thereby indirectly enhancing NP delivery to targeted cells. For instance, hydrophilic polymers added to NPs can bind to water molecules, which generates a shield around the NPs and reduces identification by immune cells.^[^ ^95^ ^]^ This approach, intertwined with immune system functions, finds better suitability in in vivo models, demonstrating enhanced NP circulation time and uptake via reduced clearance.^[^ ^96^ ^]^ Besides, “don't eat me” signals such as CD47, programmed cell death ligand 1 (PD‐L1), and CD24 are usually overexpressed in cancer cells that are capable of evading clearance by macrophages.^[^ ^97^ ^]^ Monoclonal antibodies that antagonize the “don't eat me” signals have exhibited promising potential in several cancers. NPs functionalized with CD47‐ and Integrin α4/β1 equipped macrophage membranes were thus used to intravenously deliver colchicine to atherosclerotic plaques by effectively evading phagocytosis in a murine model.^[^ ^98^ ^]^

Despite the variabilities in different experimental conditions, the improved biocompatibility of NPs introduced by surface conjugations has been widely proven. Due to the specificity of NPs recognition, the ligands bonded to NPs are generally designed to adapt to specific cell types rather than being universally applicable in different cells.^[^ ^94^ ^]^ Meanwhile, sufficient distance between ligands is desired to reduce the steric hindrance and to bind a sufficient number of ligands, thus spacers are recommended in cases using small molecules to stave off the ligands from the NP surface.^[^ ^99^ ^]^ Besides, the conjugation is conducted independently according to the various ligands and particle types, which could be influenced by environmental conditions.^[^ ^100^ ^]^ Successful NP delivery systems necessitate meticulously designed ligands with strong binding capability and stability, which can be assessed for biocompatibility and toxicity through suitable in vitro and ex vivo human‐derived models. Moreover, demonstrating the adaptability and robustness of targeting strategies identified in vitro or ex vivo systems often requires in vivo animal experiments.

## 4 Effect of Physicochemical Properties of NPs on Cellular Uptake

### 4.1 Size of NDC

It has been widely known that the size of NPs is one of the most crucial factors that affect the efficiency and pathways of cellular internalization, as well as the toxicity of living cells. When particles diffuse to or sediment at the surface of the cell membrane, the physical interaction between the NPs and cells leads to particle aggregation and clustering, subsequently inducing differential cellular responses depending on the NPs size.^[^ ^13^ ^]^ For NPs with a diameter under 200 nm, the main pathway for cellular uptake is clathrin‐ or caveolin‐mediated endocytosis, and the size of caveolae limits the internalization of larger particles.^[^ ^14^ ^]^ These particles at 500 nm to 3 µm, enter cells via phagocytosis, which is not relying on any intermediates but is a direct active response by cells.^[^ ^15^ ^]^

While numerous studies have explored the link between particle size and uptake pathways, no consistent conclusion has emerged, likely due to the diverse conditions of NDC systems and selective culture environments. A key concern is the observed agglomeration of particles in vitro and in vivo, impacting initial NP size and subsequent internalization pathways.^[^ ^15^, ^101^ ^]^ Besides, the complexity of NDC systems also introduces challenges in controlling the absolute size of NPs without alteration of other characteristics. Previous studies have investigated the interference of cellular uptake of NPs introduced by the aggregation, for example, Albanese et al. mimicked the natural formation of aggregates of AuNPs in biological conditions using Hela and A549 cells, and found that the non‐aggregated NPs exhibited better performance of cellular internalization than aggregated NP.^[^ ^102^ ^]^ Their result also reported an unpredictability of the endocytosis of NPs with similar sizes, due to the variant surface curvature and irregular shapes between particles which results in the different densities of targeting moieties, asymmetrical structure, and the binding avidity between receptor and ligands on the aggregates.^[^ ^102^ ^]^ Recently, Liu et al. have demonstrated that the heterogeneous surface charge distributions due to different surface chemistry may contribute to the aggregation behavior of the particles.^[^ ^103^ ^]^ Kenzaoui et al. have observed caveolae‐mediated endocytosis in well‐dispersed silica NPs but discovered a predominant micropinocytosis for aggregated NPs.^[^ ^104^ ^]^ While various complex models have been utilized to explore the relationship between nanoparticle (NP) size and potential functional pathways, it is generally observed that particles with a diameter in the range of 30–50 nm tend to demonstrate the most efficient internalization in different studies.^[^ ^105^ ^]^ The quest for the ideal NP size for efficient delivery remains challenging due to the intricate interplay of parameters like size, shape, and surface chemistry. This complexity hinders predictions regarding the NP biological outcomes, especially when multiple physicochemical properties are altered simultaneously.^[^ ^106^ ^]^ However, systematic investigations into the NP internalization mechanisms with diverse sizes and other physicochemical parameters hold promise for advancing nanomedicine applications.^[^ ^107^ ^]^

### 4.2 Shape of NDC

In addition to the size, the shape of the NDC has also been demonstrated to be associated with cellular internalization efficiency. In most studies, the consensus is that rod‐shaped NPs are superior to spherical NPs in cellular uptake. For example, cellular uptake efficiency has been compared among polystyrene NPs with different shapes, including rod‐, sphere‐, and disc‐shaped ones, and the results showed a higher uptake rate of rod‐ and disc‐shaped particles rather than spheres.^[^ ^108^ ^]^ The cellular internalization rate of hexagonal prism, oblong, spherical, and hollow‐core mesoporous spherical and rod‐shaped silica NPs in PC3 prostate cancer cell lines Hela and A549 cells were compared, in which the hexagonal prism rod‐shaped ones displayed the highest uptake efficiency.^[^ ^109^ ^]^ One possible explanation is the correlation between the cellular uptake efficiency and the aspect ratio of NDC, which is defined as the ratio of length to width of NP.^[^ ^110^ ^]^ Liu et al. demonstrated that with the increasing aspect ratio of particles, it becomes more difficult for cell membrane to wrap the NPs, which partially explained the association between aspect ratio and internalization.^[^ ^111^ ^]^ Shen et al. found that the oblate ellipsoids are likely to contact cell membranes with its long edge, which might lead to difficulties for cells to swallow the NPs and resulting in incomplete wrapping.^[^ ^112^ ^]^ Besides, the necessary orientation change for rod‐shaped particles during wrapping requires increased energy consumption, which in contrast decreases its cellular uptake.^[^ ^113^ ^]^ Consistently, another study also proved that the aspect ratio of rod NPs is positively correlated with internalization force and time, while the energy analysis suggested that intermittent rotation from the horizontal to vertical direction can reduce energy dissipation during the internalization process.^[^ ^114^ ^]^ To overcome the energy barrier of internalization, increasing both the number and rotation angle correlates with higher aspect ratios.

Scientific efforts have been greatly focused on probing the mechanism of cellular internalization of particles with different shapes. It has been proved that the uptake efficiency was associated with biotin conjugation, which increases the internalization of rod particles 3 times and disc‐/sphere‐ NPs two times.^[^ ^108^ ^]^ Possible explanation could be attributed to the larger surface‐to‐volume ratio of rod particles, which enables more active presenting of the surface sites to biotin conjugation, allowing the increased interaction between cells and NPs for enhanced uptake. Besides, the early‐stage particle uptake depended on the geometry of NPs, and the pathways of particles with different shapes differed a lot according to inhibitor experiments. Particles with sharp edges were demonstrated to have suppressed endocytosis.^[^ ^12c^ ^]^ A computerized molecular dynamics simulation was developed for comparing the possible difference in NPs migration efficiency through lipid membranes, which revealed a significant variation among NPs with different shapes, including rod‐, cube‐, sphere‐, cone‐, and pyramid‐ shaped particles, suggesting the influence that NPs shape may have on their interaction with cell membranes.^[^ ^115^ ^]^ In addition, a dissipative particle dynamics simulation approach speculated the possible affection of the initial orientation and location of the NPs with different shapes, which could be regarded as an acceptable explanation for the variation of penetration efficiency of NPs.^[^ ^116^ ^]^

### 4.3 Surface Charge

The charge of NDC surface directly affects the electrostatic interaction between NDC and cell membranes.^[^ ^117^ ^]^ Due to the negative charge of the cell membrane, it displayed enhanced attraction to positively charged NPs instead of neutral and negative ones.^[^ ^118^ ^]^ Compared with uncharged NPs, the cationic counterparts induce structure disorder of the lipid bilayer at the adhesion location, and exhibit activated thermo‐dynamical interaction and improved membrane‐wrapping with the cells. Other studies also suggest membrane fluidity was induced by positively charged particles, while negatively charged particles led to gelation of the bilayer, all of which results in the various internalization of NDC with different ionization properties. On the contrary, neutral NPs do not induce gelation of the cell membrane but may impair the penetration process, lowering the cellular uptake efficiency compared to negatively charged NPs. Moreover, it has also been speculated that positively charged NPs may disrupt the integrity of the cell membrane, thus achieving enhanced internalization,^[^ ^119^ ^]^ leading to increased cell toxicity and cell death. Neutralization of the cells followed by the interaction between cationic NPs and negatively charged cell membrane leads to depolarization and calcium influx, which modulates intracellular pathways resulting in inhibited proliferation and reduced cell viability.^[^ ^120^ ^]^

The mechanism of cell penetration and delivery also differs a lot among NPs with different charges. Cellular uptake of positively charged particles mainly relies on micropinocytosis, and negatively charged NPs are taken by the cells through clathrin‐/caveolae‐independent endocytosis.^[^ ^12^, ^13^, ^113^ ^]^ Cho et al. reported as sole path, endocytosis for the internalization of anionic and neutral NPs, whereas cationic AuNPs disrupt cell membranes and generate holes for diffusion into cells.^[^ ^121^ ^]^ A positive correlation between membrane disruption of lipid bilayer with the charge density of NPs was observed, which may be caused by the NP orientation at the initial contact with the cell membrane.^[^ ^122^ ^]^ Remarkable organ‐selective lipid NDC delivery and gene editing in vivo was achieved through the addition of cationic or anionic molecules. Specifically, 50% cationic DOTAP‐lipid NDC redirected NP/cargo delivery from the liver to the lung, while 30% anionic 18PA‐lipid NDC targeted the spleen.^[^ ^123^ ^]^ These features inspire the necessity of searching for the optimal charge and suitable molecules, which enables both efficient NDC uptake efficiency and preservation of cell membrane integrity.

### 4.4 Hydrophobicity

Hydrophobicity, another essential factor, determines the interaction between NDC and cell membranes.^[^ ^117^ ^]^ Hydrophilic NPs were demonstrated to favor adsorption on the surface of the lipid bilayer, which induces the wrapping by the membrane and subsequent endocytosis, while hydrophobic NPs can insert into the cell membrane and reach a thermodynamical balance with the hydrophobic bilayer core without causing leakage.^[^ ^124^ ^]^ Consistent with this phenomenon, Curtis et al. also observed the embedding and penetration of hydrophobic NPs into the bilayer core, while hydrophilic NPs were attached to the surface of the membrane and wrapped by a lipid bilayer.^[^ ^125^ ^]^ Lee et al. investigated the activity of AuNPs with mixed hydrophobicity ligands when exposed to liposome membrane, and the hydrophilic ligands were observed to retain on the surface and interact with the aqueous solution, while the hydrophobic ligands were immersed in the hydrophobic core of the bilayer.^[^ ^126^ ^]^

However, hydrophobicity has been suspected to work together with particle size for the determination of the NPs behavior after interacting with the cell membrane. A simulation approach was developed by Curtis et al, which showed that not all the hydrophilic NPs undergo wrapping. NPs larger than 2 nm were packaged for phagocytosis, and those smaller than 1 nm were inserted directly into the aperture of the bilayer, interacting with the hydrophilic groups exposed by cytomembranes.^[^ ^125^ ^]^ Jing et al. demonstrated that the pore formation reaction of hydrophobic polystyrene NPs on phosphatidylcholine was unrelated to the size of particles, and the hydrophobic interaction determined by ionic strength was crucial for adsorption and wrapping reaction.^[^ ^127^ ^]^

### 4.5 Elasticity

Elasticity, encompassing rigidity, hardness, and stiffness, plays a crucial role in governing the efficacy of NDC cellular internalization. Particle elasticity is evaluated using techniques like atomic force microscopy, nanoindenter, and rheometer to measure Young's modulus. Higher Young's modulus scores, seen in QDs, metal NPs, and magnetic NPs, indicate greater elasticity and more efficient cellular uptake than lower Young's modulus particles like polymers, liposomes, and hydrogels. It has been suggested that the softer NPs require higher energy consumption during the process of wrapping. Efforts have been pursued for exploring the potential influence of elasticity on NP‐cell interactions. According to a molecular dynamics simulation developed by Shen et al., the thermodynamic driving force competes with receptor diffusion kinetics in endocytosis to reach sufficient free energy for the wrapping of NPs, during which the elastic NPs require more energy for elastic deformation and thus result in a less efficient cellular internalization.^[^ ^128^ ^]^ Sun et al. revealed a rigidity‐dependent cellular uptake according to simulations at the atomistic level, in which rigid NPs exhibited easier access through the cytomembrane.^[^ ^129^ ^]^ Palomba et al. demonstrated a short‐lived (<30 s) interaction between soft polymeric nanoconstructs and macrophages, which is insufficient for effective recognition of NPs, suggesting a diminished internalization capacity of by macrophages.^[^ ^130^ ^]^

However, the findings are not consistently uniform. Yu et al. recommended poly(lactic‐co‐glycolic acid) (PLGA) core‐lipid shell NPs with moderate rigidity to optimally penetrate biological hydrogels, while rigid NPs and overly soft NPs were impeded due to failed deformation or interactions with the hydrogel network.^[^ ^131^ ^]^ Chen et al. built a model with deformable elasticity and tunable morphology of NPs and demonstrated that the elastic NPs internalization was determined by their shape: cellular uptake efficiency was significantly influenced by deformational properties in spherical NPs, while the changeable shape could promote the uptake of oblate NPs.^[^ ^132^ ^]^ Besides, an in vivo model revealed that both neoplastic and non‐neoplastic cells exhibited favorable uptake of soft nanolipogels, and the soft NPs accumulated more in tumors whereas the elastic ones were retained in the liver.^[^ ^133^ ^]^ Despite the lack of consistent correlation with elasticity, it could be considered a valuable parameter for particle design aimed at improving cellular penetration and tumor delivery efficiency.

Lipid NDCs play a pivotal role in drug and nucleic acid (i.e., siRNA and mRNA) delivery both in vitro and in vivo, typically encompassing various lipids like cationic/ionizable lipids, phospholipids, cholesterols, and PEG‐lipids. The structural lipids and PEG‐lipids significantly influence the stability, size, and blood biocompatibility of NDC.^[^ ^134^ ^]^ Recent studies further demonstrated that modifying the molecular structure of lipids, like cholesterol derivatives in lipid NDC, can impact NP membrane rigidity, curvature, and deformability, leading to improved gene delivery efficiency.^[^ ^134^, ^135^ ^]^ Through analysis of over 2,000 in vivo drug delivery data points, Paunovska et al. demonstrated that lipid NPs with esterified cholesterol were more effective in delivering nucleic acids in both wildtype and transgenic mouse models than those with regular or oxidized cholesterol.^[^ ^136^ ^]^ Notably, cholesteryl oleate infused lipid NPs efficiently delivered siRNA and sgRNA to liver endothelial cells in vivo, outperforming unmodified cholesterol lipid NPs.^[^ ^136^ ^]^ Also, improved gene transfection up to 25‐fold enhancement in cell lines is observed when incorporating C‐24 alkyl phytosterols into lipid NPs compared to cholesterol‐lipid NPs.^[^ ^137^ ^]^ The lipid NPs crafted with C24 alkyl derivatives of cholesterol (e.g., *β*‐sitosterol, fucosterol, campesterol, and stigmastanol) exhibiting a polymorphic shape along with varying degrees of multilamellarity and lipid partitioning had substantial morphological differences compared with spherical lipid NPs.^[^ ^135^ ^]^ Engineering lipid NPs consisting of PEG‐lipid and *β*‐sitosterol were also found to boost the intracellular delivery of mRNA into lung tissues via inhalation in a mouse model.^[^ ^138^ ^]^ In addition, oxidative modifications on the cholesterol tail enhance the accumulation of lipid NPs in liver endothelial cells and Kupffer cells rather than hepatocytes,^[^ ^139^ ^]^ and cholesterol‐amino‐phosphate (CAP) lipids as effective vehicles for delivering RNA, including self‐amplifying RNA, to spermatocytes, addressing male infertility caused by a specific genetic mutation.^[^ ^140^ ^]^ The addition of cholesterol derivatives/modifications in lipid NDC is a good example to regulate NDC rigidity as well as membrane integrity, which improves NP stability and transfection efficiency in delivering genetic materials.^[^ ^134a^ ^]^

## 5 Hierarchical Targeting Technologies for Precision NP Cellular Delivery In Vivo

When the grand subject of the NDC delivery platform develops from the cellular level to the whole‐body system, intracellular accumulation, and retention of NDC raise new challenges for the highly controllable development of nanotechnology. Enhanced cellular internalization of NDC is often achieved through different surface charges or modified targeted ligands and subsequent improved interactions between particles and cell membranes. However, synchronous efficient cellular internalization and accumulation are difficult to achieve. The positively charged NDC are associated with better particle‐cell interactions and accelerated ingestion while displaying higher sensitivity in recognition and clearance.^[^ ^141^ ^]^ Neutral‐ or negatively charged particles achieve successful intracellular accumulation through EPR effect but worse particle‐cell interaction and ineffective internalization.^[^ ^13^, ^15^ ^]^ Therefore, to achieve both high cellular uptake and retention for NDC platforms, it is essential to develop changeable properties of the NPs that meet several requirements in different microenvironments when processing through different cells/tissues/bodies.^[^ ^142^ ^]^

### 5.1 Switchable Surface Charges

As the examples mentioned above, the surface charge of the particles is one of the main obstacles preventing simultaneous cellular internalization and accumulation. Thus, the design of particles with switchable surface charges becomes a powerful and useful solution. Existing strategies for improving blood circulation and tumor penetration are mainly focused on developing negatively or neutrally charged NPs such as PEG‐coated NPs, and positive charges are highly recommended for enhancing interaction between NPs and negatively charged cell membranes once NPs arrive at the surface of tumor tissues. In combination with the fact that the extracellular environment of the tumor tends to exhibit lower pH and a weak acidic condition owing to the increased glycometabolism and subsequent lactic acid fermentation, a variety of pH‐triggered strategies have been developed. 2,3‐dimethylmaleic anhydride (DMMA) has been introduced as the shell of positively charged NPs.^[^ ^143^ ^]^ DMMAs carboxyl groups are designed to wrap particles through the amide bonds to amino groups at the NP surface, and thus the particles exhibited negative charges in the physiological environment. However, the amide linkage between DMMA and amino groups easily cleaves in an acidulous tumor environment, which exposes the protonated amino groups and leads to the reversion of surface charge. Du et al. have reported improved cellular internalization of this kind of charge‐switchable particles in tumor tissues, demonstrating a remained negative surface charge of NPs at pH 7.4 and different distribution of NPs in the cytoplasm at pH 6.8.^[^ ^144^ ^]^ In addition to the bonds between carboxyl and amino groups, electrostatic adsorption has also been introduced as another strategy for the linkage between NPs and the negatively charged coating shield.^[^ ^145^ ^]^ Solomun et al. constructed a multi‐component polymer system consists of cationic and hydrophobic particles with high delivery performance, a pH‐responsive block copolymer as shielding system, and an alternative to PEG for enhanced biocompatibility and circulation time. This cationic NDC achieved excellent stability at extracellular conditions (pH 7.4) and high transfection efficiency, as well as membrane leakage at acidic conditions (pH 6).^[^ ^146^ ^]^ Besides, Guan et al. also designed a PEI‐based gene carrier system loading therapeutic nucleic acid, which was tightened using PEG through the bonds between the aldehyde of PEG and amino of PEI.^[^ ^147^ ^]^ This system was triggered by an environment change of pH and responded to acidic conditions rapidly, allowing the detachment of the PEG shield and leading to subsequently enlarged size and surface charge transformation. According to the charge/size double‐reverse system, the carriers are easy to implement prolonged blood circulation and increased uptake efficiency by tumor cells.^[^ ^147^ ^]^

**Figure 7:**
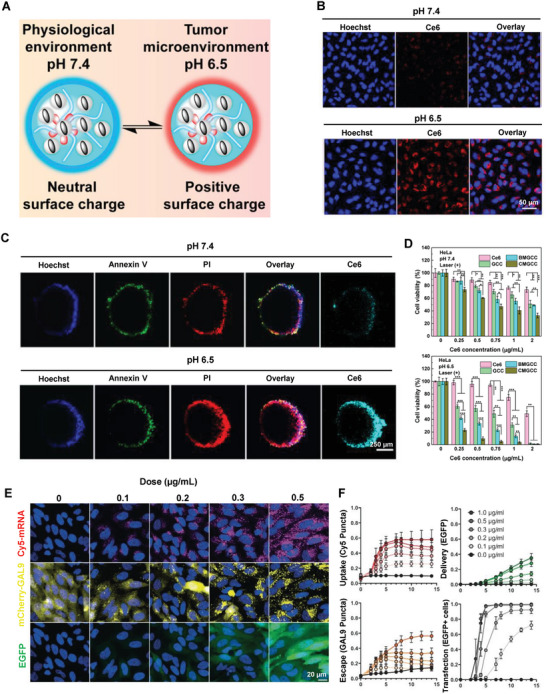
A) Schematic illustration of pH‐triggered surface charge switch strategy using the chlorin‐e6‐modified glycol chitosan (GC) polymeric micelles with pH‐sensitive surface charge switchable from neutral to positive. Reprinted with permission,^[^ ^149^ ^]^ Copyright 2021, American Chemical Society. Confocal fluorescence images of Hela cells (B) and CLSM images of 3D multicellular spheroids (C) after treatment with the intelligent multifunctional synergistic nanoplatform (CMGCC) at pH 7.4 and 6.5. The cell nuclei, early apoptotic, and dead cells were stained with Hoechst, FITC‐Annexin V, and PI, respectively. D) Cell viability analysis illustrating the tumor cell killing ability of CMGCC compared to other systems,^[^ ^149^ ^]^ Copyright 2021, American Chemical Society. E) Live‐cell microscopy of mCherry‐GAL9 HeLa cells subjected to varying MC3‐LNPs doses (0–0.5 µg mL^−1^) at 10 h post‐dosing. (F) Quantitative analysis of the dose response in HeLa cells harboring the mCherry‐GAL9 reporter was conducted within 14 h at concentrations of 0–1 µg mL^−1^ MC3‐LNPs. The measurements encompassed LNP uptake (Cy5‐mRNA structures), endosomal escape (mCherry‐GAL9 structures), mRNA delivery (EGFP fluorescence), and transfection (EGFP positive cells). Reprinted with permission,^[^ ^157a^ ^]^ Copyright 2021, Springer Nature.

The functional surface groups of surface charge‐switchable particles include amines, morpholine, and histidine, which often respond to weak‐acidic conditions in the tumor environment and participate in protonation, leading to the reversion of NP surface charge and subsequent intracellular accumulation. Qiao et al. introduced a poly (b‐amino ester)‐based copolymer as the carrier of hydrophobic anticancer drugs, which remains stable in neutral conditions but labile to get the hydrophobic/hydrophilic equilibrium disrupted in an acidic environment, and thus achieves enhanced cellular uptake and drug release.^[^ ^148^ ^]^ Zhu et al. have developed an intelligent multifunctional synergistic nanoplatform (CMGCC) encapsuled in chlorin‐e6‐modified glycol chitosan (GC) polymeric micelles, with pH‐sensitive surface charge and capable to switch from neutral to positive^[^ ^149^ ^]^ (**Figure** **7**A). Briefly, this system was designed to deliver photosensitizers into tumor cells and improve the efficacy of photodynamic therapy. At the pH of 6.5, both in vitro and in vivo experiments demonstrate an enhanced internalization of CMGCC into HeLa cells and subcutaneous HeLa tumors, which improved the efficacy of photodynamic therapy with significant cancer cell killing ability^[^ ^149^ ^]^ (Figure 7B–D).

The transformation from zwitterionic to cationic triggered by the change in pH environment also provides feasibility into the surface charge‐related hierarchical response. Options of pH‐responsive zwitterionic groups include phosphorylcholine,^[^ ^150^ ^]^ carboxybetaine,^[^ ^151^ ^]^ alkoxyphenyl acylsulfonamide,^[^ ^152^ ^]^ lignin,^[^ ^153^ ^]^ and glycol chitosan.^[^ ^149^ ^]^ A gold NPs formula was attempted to get reconstructed using aryl acylsulfonamide groups, which exhibited positive surface charge and significantly enhanced cellular uptake at acidic pH, while alkyl acylsulfonamide group coated particles retained a zwitterionic surface and unsatisfied cellular internalization.^[^ ^152^ ^]^ Modification of the NP surface using mixed charge species in specific ratio could also achieve zwitterionic surface characteristics and different pKa values within a certain range.^[^ ^154^ ^]^ A gold nanostar (GNS) was modified using amine/carboxyl‐terminated PEG by different ratios, and the pKa value exhibited continuous correlation to the number of amine/carboxyl PEGs, among which a sort of GNS displayed significantly different toxicity to Hela cells, which might be attributed to the highest intracellular efficiency of the GNS resulted from the modified zwitterionic characteristics.^[^ ^120^ ^]^

Changeable surface charge is widely exploited for facilitating endosomal escape and cytosolic cargo release in cellular delivery of NDC. This is exemplified using cationic/ionizable lipids for oligonucleotide delivery. Ionizable lipids used in lipid NDC attain positive charge in acidic endosomes while remaining neutral in circulation,^[^ ^134b^ ^]^ enhancing in vivo delivery by reducing interactions with blood cell membranes and improving NP biocompatibility.^[^ ^134^, ^155^ ^]^ Within endosomes, these lipids acquire positive charge due to lower pH, destabilizing membranes and promoting NP escape and cargo release into the cytosol, enhancing DNA transfection and mRNA delivery.^[^ ^134^, ^155^ ^]^ The Galectin (GAL) protein family, characterized by cytosolic expression and carbohydrate recognition domains (CRDs) binding to β‐galactoside sugars, is harnessed to visualize endosome rupture in living cells.^[^ ^156^ ^]^ Galectins (GAL8/9) are attracted to endosomes when inner‐leaflet β‐galactosides become exposed due to membrane damage, a process observed during pathogen infections and induced by lipid and polymer NDC, facilitating nucleotide cargo release.^[^ ^157^ ^]^ An advanced imaging platform has been developed to simultaneously track cellular uptake, endosomal escape, and mRNA translation during lipid and polymeric NDC delivery to multiple mCherry‐GAL9 reporter cell lines.^[^ ^157a^ ^]^ Cy5‐labeled, EGFP‐encoding mRNA lipid NDC (MC3‐lipid NPs) were formulated, demonstrating dose‐dependent effects on cellular uptake, endosomal escape, and mRNA delivery and transfection by quantitatively assessing Cy5, mCherry, and EGFP fluorescence in Hela cells (Figure 7b). Additionally, lipid NDC with pH‐switchable zwitterions and hydrophobic tails adopt cone shapes within endosomes, prompting membrane transformation and escape. This formulation, utilizing zwitterionic ionizable lipids, enhances endosomal escape, leading to increased protein expression and genome editing in vivo.^[^ ^158^ ^]^ Detailed descriptions of lipid NDC design and their application for delivering genetic materials like mRNA and siRNA are not covered here, as they are addressed in previous reviews.^[^ ^134^, ^155^, ^159^ ^]^

### 5.2 Changeable Particle Sizes

The dynamic size of NDC is one of the most critical factors that determine its cellular uptake and retention. The NDC with a diameter of less than 400 nm can extravasate from leaky vasculature into tumor interstitium,^[^ ^160^ ^]^ and those ranging from 10 to 100 nm were generally accepted with reduced liver capture and renal filtration, which could be generally applied in clinical settings with lower hepatotoxicity and nephrotoxicity.^[^ ^142^ ^]^ Small‐sized NDC displays better penetration efficiency into tumor but are easily to re‐enter blood circulation, while large NDC may exhibit improved ability to retain in the cytoplasm or even in nuclei of tumor cells but cannot be captured by target cells efficiently.^[^ ^161^ ^]^ Hence the possibility of designing the changeable size of the particles appears as a bold assumption in the stage of NP application. The core idea of the changeable particle size development is stimulation. Possible strategies include stimulation‐triggered aggregation, size decrease, and supermolecular co‐assembly.

The microenvironment of tumor cells triggered NP aggregation is one of the widely explored strategies to achieve simultaneous cell internalization and intracellular retention.^[^ ^162^ ^]^ A size‐changeable nanotheranostic agent, composed of polyprodrug‐modified iron oxide NPs and an aggregation‐induced emission photosensitizer, is developed for MRI‐guided chemo/photodynamic combination therapy. These nanotheranostic agents initially have a diameter of approximately 90 nm, enabling passive tumor tissue targeting, followed by aggregation in the acidic tumor microenvironment for enhanced retention and MR signal enhancement.^[^ ^162a^ ^]^ Human serum albumin (HAS) has been reported to be engaged as a biocompatible NDC loading doxorubicin (DOX), and the HAS‐DOX complex was modified with protonation of carboxylic groups to enhance the hydrophobicity, allowing for the aggregation of the complex in tumor microenvironments. An in vivo study has demonstrated its prolonged blood circulation and improved tumor retention by Zhang et al.^[^ ^163^ ^]^ Besides, a liquid metal NP has also been reported to aggregate for larger NP construction, which enables developed tumor accumulation and drug release.^[^ ^164^ ^]^ Wong et al. reported a platform constructed by 10 nm QDs encapsulated in 100 nm gelatin. The gelatin shield degraded after being transferred into tumor tissues, and the released QDs exhibited improved cellular penetration superior to the large complex.^[^ ^165^ ^]^ Besides, microenvironment‐activated aggregation is combined with light irradiation to achieve re‐dispersion of small‐sized particles, which exhibited enhanced tumor retention and subsequent fast elimination to reduce the risk of toxicity.^[^ ^166^ ^]^

**Figure 8:**
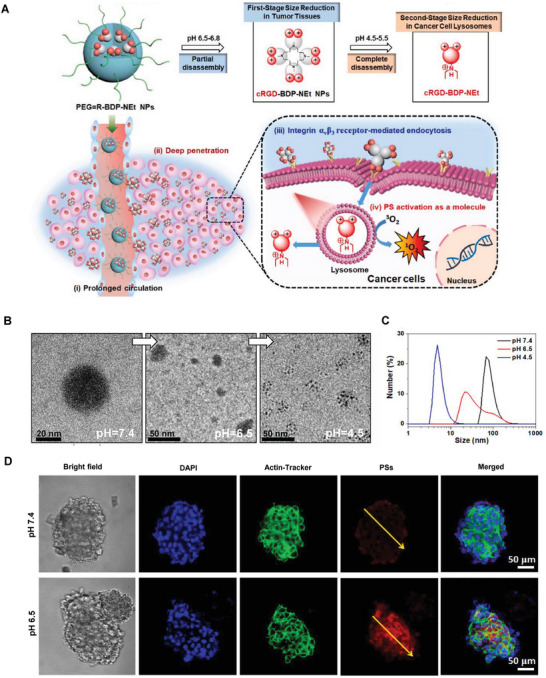
A) Schematic illustration for hierarchical disassembly of PEG = R‐BDP‐NEt NPs for enhanced tumor penetration. Upon intravenous injection, the NPs exhibit prolonged circulation time in blood (i). The extra‐cellular microenvironment (pH ≈6.5) in the tumor induced the first‐stage size reduction between PEG and cRGD peptide, enabling enhanced accumulation and deep penetration in tumor (ii). In addition, the cRGD peptides exposed on the NP surface could enhance the specific recognition of the NPs by cancer cells that overexpress integrin ανβ3 receptors (iii). Then the more acidic pH in lysosomes (pH ≈4.5) triggers the second‐stage size reduction of NPs, leading to complete disassembly of the NPs into single cRGD‐BDP‐NEt molecule. B,C) TEM images (B) and hydrodynamic size distributions (C) of the PEG = R‐BDP‐NEt NPs at different pH conditions. D) CLSM images of MDA‐MB‐231 cell spheroids for the penetration test. The cell spheroids were incubated with PEG = R‐BDP‐NEt NPs at pH 7.4 and 6.5 for 3 h, respectively, showing a better tumor penetration of NPs (red) at pH 6.5 conditions. Reprinted with permission,^[^ ^167^ ^]^ Copyright 2021, Elsevier B.V.

To avoid rapid renal clearance of NPs, some particles were designed to be oversized to achieve longer blood circulation. Meanwhile, the large size of NPs will lead to ineffective cellular internalization. To address both a prolonged circulation and efficient delivery into tumor cells, which requires appropriate sizes of NPs at the initial entering the body, but to achieve size reduction after reaching the tumor tissue to enhance uptake by tumor cells^[^ ^167^ ^]^ (**Figure** **8**). Wang et al. developed self‐assembly‐based platinum/poly(amidoamine) (Pt/PAMAM) conjugated to polycaprolactone (PCL) and coated with PEG. The core Pt/PAMAM (about 5 nm) are amplified to about 100 nm with PCL and PEG coating, which shows advantages in blood circulation but will be released from the complex by pH‐sensitive activation and realize better cellular uptake in tumor extracellular environment.^[^ ^168^ ^]^ Other approaches have also been proposed regarding the changeable size of particles such as a novel nano‐complexes composed of lipid‐PEG and spiropyran, which was found to exhibit photoswitching features that induced shrinkage from 103 nm to 49 nm when exposed to irradiation at 365 nm. In addition advantages in tissue penetration have been shown in a subcutaneous mouse model.^[^ ^169^ ^]^

### 5.3 Surface Ligands Activation

In addition to the transformation of the surface charge and particle size, activation of NDC surface ligands has been investigated as another tactic for escaping renal excretion and improved interaction with tumor cells. Ligands conjugated to NDC are often cancer‐specific and permit the mutual recognition of both NPs and tumor cells, allowing for receptor‐mediated endocytosis. Possible ligands that are explored in previous research include monoclonal antibodies, peptides, and other small molecules, promoting the NPs to stick on tumor cells and triggering subsequent responses.^[^ ^5^, ^170^ ^]^ However, ligands conjugated to the particles might affect the stealth property of NDC, which could induce their elimination by macrophage or unintended uptake by normal tissues, and thus raise several challenges for further applications.^[^ ^171^ ^]^

**Figure 9:**
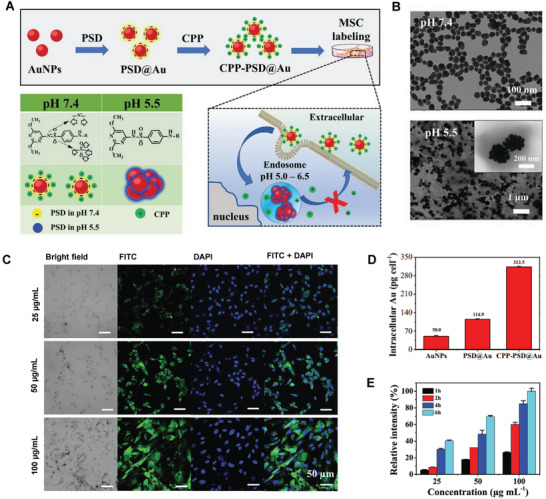
A) Schematic illustration of a pH‐sensitive Au nanotracers (CPP‐PSD@Au) fabricated by sequential coupling of AuNPs with sulfonamide‐based polymer (PSD) and cell‐penetrating peptide (CPP). B) TEM images of the CPP‐PSD@Au at pH 5.5 and 7.4, respectively. C) Laser confocal microscopy images of mesenchymal stem cells labeled with CPP‐PSD@Au at different concentrations for 4 h. D) Amount of intracellular AuNPs, PSD@Au, and CPP‐PSD@Au in mesenchymal stem cells after incubation at 0.1 mg mL^−1^ for 4 h. E) Relative intensity of mesenchymal stem cells incubated with CPP‐PSD@Au at various concentrations for different time points. Reprinted with permission,^[^ ^176c^ ^]^ Copyright 2021, Wiley‐VCH.

To solve these problems, sheddable surfaces constructed by long polymer chains connecting the NPs are designed to avoid the recognition by normal cells as well as to reduce the degradation of the molecules by enzymes in the blood‐circulation. PEG has contributed to several platforms as a shield for protecting the ligands including RGD peptides,^[^ ^172^ ^]^ TAT peptides,^[^ ^173^ ^]^ lysine,^[^ ^174^ ^]^ and other molecules. The PEG shell bonded to NPs through pH‐sensitive hydrazine in physiological conditions and turned off the targeting function of the ligands, while it can be detached from the NPs in acidic tumor tissues so that the surface ligands can be exposed to tumor receptors triggering targeted recognition.^[^ ^175^ ^]^ Cell‐penetrating peptides (CPPs), typically consist of 6–30 residues, including HIV TAT peptide, penetratin, oligoarginine, transportan, and TP10, are widely coupled in several organic and inorganic NDC systems (such as gold, silver, iron oxide, polymeric, and lipid NPs) for delivering genetic materials to cells in vitro or enhancing tumor penetration and cellular upake in vivo.^[^ ^176^ ^]^ An innovative gene delivery NDC system by combining octaarginine (R8), a CPP, and YSK05, a pH‐sensitive cationic lipid was developed to synergic enhance transfection efficiency and endosomal escape of the DNA in vitro.^[^ ^177^ ^]^ Incorporation of a hydrophobic moiety into arginine‐rich cell‐penetrating peptides (CPPs), such as N‐Terminal acylation of oligoarginines (stearyl‐octaarginine, STR‐R8), was observed to enhance the delivery efficiency of plasmid DNA (pDNA) and siRNA.^[^ ^176b^ ^]^ Targeted delivery of pDNA to lung endothelium was further achieved in a mouse model using STR‐R8‐modified lipid NDC.^[^ ^178^ ^]^ Also, PEG, polypeptides, and macromolecules can be conjugated to NDC for shedding unselective interaction of CPPs before entering tumors and improving the targeted capability. For example, a PEG‐*Dlink_m_*‐R9 polymeric NDC was designed to achieve prolonged circulation, enhanced tumor cell accumulation, and thus improved inhibition of non‐small cell lung cancer growth.^[^ ^179^ ^]^ Owing to the rapid response to specific enzymes in the tumor extracellular environment, these shells are ready to be degraded and the core ligands are then exposed to specific receptors enhancing NDC delivery. For example, hyaluronic acid (HA) has been reported to protect CPP‐modified liposomes and displayed a negative charge with superior blood‐circulation. Responding to HAase, direct exposure of CPP‐liposomes followed by degradation of the HA shell leads to better cellular internalization.^[^ ^180^ ^]^ Yu et al. has reported a pH‐sensitive Au nanotracer (CPP‐PSD@Au) fabricated by sequential coupling of AuNPs with sulfonamide‐based polymer (PSD) and cell‐penetrating peptide (CPP). This system achieves variable charge by PSD and improved biocompatibility by CPP, which encounters self‐aggregates and disengagement of CPP after entering the tumor cells under the acidic conditions, enabling satisfactory cellular internalization and increased intracellular retention^[^ ^176c^ ^]^ (**Figure** **9**). Sun et al. also developed a cleavable coating layer‐based platform (iron oxide NPs) for monitoring enzyme activity in tumors.^[^ ^181^ ^]^ These coating layers granted the water‐soluble characteristic of the particles and were ready to be moved by specific enzymes, which exposed the initial surface of the particles and achieved selective activation of the nanoprobes. The results showed that the modified NDC cause significantly reduced adverse effects compared with the traditional ones, which suggested the promising potential of the enzyme‐based grafting/peeling‐off strategy for tumor imaging.

## 6 Novel Physiological Models Mimicking the In Vivo NP Cellular Delivery

The goal of the NDC delivery platforms is to prompt their potential or utility to serve as imaging agents, drug carriers, and therapeutic components in preclinical studies and clinical practice for disease monitoring and treatment, especially for tumor management of cancer patients. As summarized in the above sections, particle characteristics including size, shape, material, surface modification, etc., contribute to intracellular delivery efficiency, which also urges evidence for customized particle design in various application scenarios. However, penetration of particles and release of drugs carried by NDC highly depend on physiological surrounding environmental parameters, such as pH, temperature, and extracellular matrix (ECM) constitution.^[^ ^182^ ^]^ For instance, in addition to malignant cells, the tumor microenvironment is composed of stroma cells, immune cells, vessels, and fiber, which form a sophisticated and complex system for supporting tumor growth and progression. Therefore, conventional 2D monolayer cell culture is insufficient for evaluating the real delivery efficiencies in physiological conditions, while in vivo models lack evidence for concrete mechanisms affecting the fate of intracellular NPs, which encourages the development of novel in vitro *and* ex vivo bio‐engineered microtissues as novel platforms mimicking in vivo NPs transportation.

We discussed the current state‐of‐the‐art models ranging from in vitro 3D multicellular tumor spheroids model, in vitro 3D organ‐specific models like the blood‐brain barrier model and air‐liquid interface inhalation model, to ex vivo organotypic models including organ‐on‐a‐chip (simplified) and precision‐cut tissue slices from animal or human tissues that could be applied in various NDC conditions satisfied with different requirement.

### 6.1 Multicellular Tumor Spheroids (MCTS) Model

**Figure 10:**
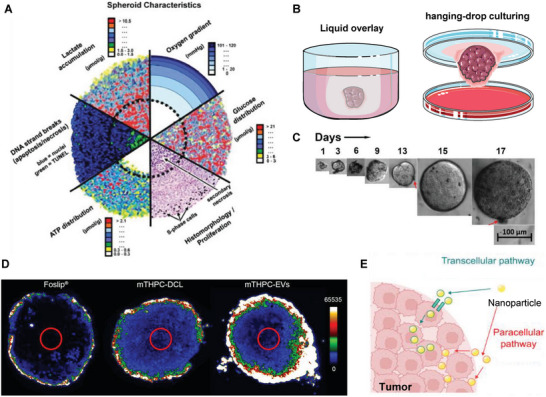
Multicellular tumor spheroids (MCTS) model. A) A schematic summary of the cells and microenvironment inside a MCTS, illustrating with high similarities to solid tumors. Reprinted with permission,^[^ ^183b^ ^]^ Copyright 2010, Elsevier B.V. B) Cell culturing strategies for generating MCTS, including liquid overlay applied by low adhesive surface coating of substrates, or hanging‐drop culturing of cell suspension on the underside of culture vessel lid. C) Bright‐filed images illustrating the development of a 3D tumor spheroid from immunomagnetically isolated cancer cells. Reprinted with permission,^[^ ^186^ ^]^ Copyright 2023, Wiley‐VCH. D) Fluorescence images of cryosections of co‐cultured spheroids (FaDu human pharynx squamous cell carcinoma and CAF granular fibroblasts) after incubated with different mTHPC‐loaded nanovesicles. Reprinted with permission,^[^ ^188b^ ^]^ Copyright 2021, Springer Nature. E) Transcellular and paracellular pathways of NPs penetration into MCTS. Reprinted with permission,^[^ ^195b^ ^]^ Copyright 2023, Wiley‐VCH.

Multicellular tumor spheroids (MCTS) are 3D structured models constructed by self‐assembly of suspended cells, which attributed with higher similarities to solid tumors than 2D monolayer cultured cells^[^ ^183^ ^]^ (**Figure** **10**A). The main principle for MCTS formations is to resist interaction between cell and substrate, and promote coupling between cells.^[^ ^183b^ ^]^ Liquid overlay applied by low adhesive surface coating of substrates,^[^ ^184^ ^]^ or hanging‐drop culturing of cell suspension on the underside of culture vessel lids,^[^ ^185^ ^]^ are the two main strategies to initialize spheroid fabrication, which stimulates cell aggregation and proliferation, resulting in the generation of 3D cultured tumor spheroids^[^ ^186^ ^]^ (Figure 10B,C). Sharing similar histological structures with solid tumors, MCTS have been dedicated to research into cell proliferation and differentiation, cell cycle, intercellular communication, and of course, therapeutic orientations in solid tumors for a long time, and was first introduced for in vitro investigation of NDC since 2004.^[^ ^187^ ^]^ With this method, various materials were investigated as drug carriers for a deeper understanding of their intracellular fate in physiological environments, such as block copolymer micelles, polymer NPs, AuNPs, and diverse inorganic NPs^[^ ^188^ ^]^ (Figure 10D). In addition, liposomes,^[^ ^189^ ^]^ lipid nanoemulsions,^[^ ^190^ ^]^ dendrimers,^[^ ^191^ ^]^ and hyper‐branched molecules also served as NDC and were widely investigated with MCTS models.^[^ ^183a^ ^]^ For instance, enhanced efficacy in delivering anticancer drugs to MCTS by pH‐responsive liposomes^[^ ^189^ ^]^ and photodynamic therapy through prolonged delivery of the photosensitizer m‐tetrahydroxyphenylchlorin utilizing Lipidots (nanoemulsions) within MCTS.^[^ ^190^ ^]^

However, despite the consensus that 2D and 3D models generate similar results in cellular uptake efficiency and toxicity measurements, underline differences between the two models need to be considered with detailed interpretation. For instance, particle penetration contributes to cellular responses in 3D models, since the structure has prevented the interior cells from direct interaction with suspended particles around, and involuntarily leads to higher uptake on the periphery of the spheroid.^[^ ^192^ ^]^ This could be more obvious, especially for particles adhering to the surface of MCTS, which might lead to intense cellular uptake but limited penetration. Evidence has been raised to support the assumption, of lower toxicity in the 3D model than in 2D culturing, which might be explained by the predominant uptake of NPs in exterior cells instead of affecting those internal of the spheroids.^[^ ^193^ ^]^ Surprisingly, besides the difference in toxicological consequences, cells in the two models have experienced distinct inflammatory responses and cell death mechanisms. Chia et al. found that ZnO NPs displayed lower toxicity in 3D colon cell spheroids than in 2D models, and the cells underwent necrosis in the 2D cell culturing while displayed apoptosis in the periphery of 3D spheroids.^[^ ^194^ ^]^ Theoretically, the penetration of NDC mainly depends on transcellular transport, or through the extracellular matrix from the periphery to the inner part of tumor tissues^[^ ^194^, ^195^ ^]^ (Figure 10E). From this perspective, 3D cultured models including MCTS are superior to 2D monolayer models, which may provide more reliable evidence for in vivo translation.

### 6.2 In Vitro Models Mimicking the Blood–Brain Barrier

**Figure 11:**
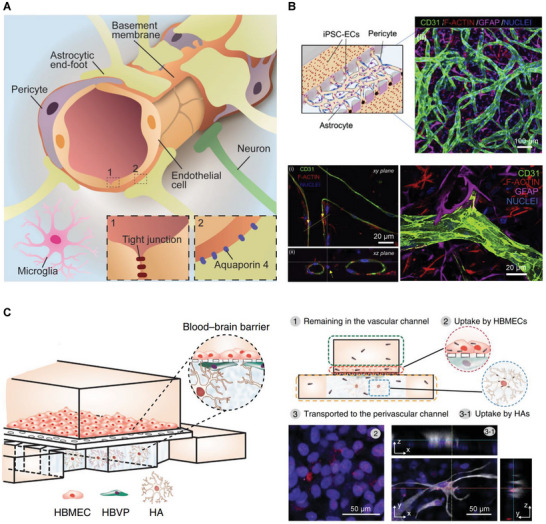
In vitro models mimicking blood‐brain barrier. A) Schematic illustration of the construction and structure of the human Blood–Brain Barrier (BBB), with tight junctions (TJs) between endothelial cells, and astrocytes contacting the microvasculature with their end‐feet and expressing polarized distribution of water channel aquaporin‐4 (AQP4). Reprinted with permission,^[^ ^197^ ^]^ Copyright 2021, Elsevier. B) A 3D BBB model replicating the microvascular network in fibrin gel using vasculogenesis strategies. Reprinted with permission,^[^ ^200^ ^]^ Copyright 2018, Elsevier. C) Another modified microengineered BBB platform with reduced reactive astrogliosis stimulating the neuroinflammation under pathological conditions, which captured 3D NP penetration and distributions at cellular levels. Reprinted with permission,^[^ ^202^ ^]^ Copyright 2020, Springer Nature.

Central nervous system (CNS) disease especially brain tumors are one of the deadliest causes in humans and nanotechnology‐enabled agents have exhibited promising potential in brain disease diagnosis and treatment.^[^ ^196^ ^]^ However, the most challenging part of nanomaterial‐based therapeutic or diagnostic agents in brains is their limited access to brain tissues due to the blood–brain barrier (BBB) thus hampering drug internalization^[^ ^197^ ^]^ (**Figure** **11**A). Conventional cell culture platforms are insufficient owing to the complexity of both CNS and BBB, which encourages use of dynamic BBB models mimicking the physiological environment as an appropriate approach prior to in vivo and clinical studies.^[^ ^198^ ^]^

Given its importance as a gatekeeper with intricate conveyance mechanism, the endothelium of the brain circulation system was the first cell type investigated in vitro BBB models. The tight endothelial barrier function was stimulated by mono‐cultured specified brain endothelial cells expressing BBB‐specific proteins, or co‐culturing of endothelial cells and astrocytes in 2D and 3D models reconstructing the BBB microenvironment with the assistance of transwell assays assessing permeability of drugs and nanocarriers, which is reproducible and cost‐effective.^[^ ^198^, ^199^ ^]^ However, the BBB morphology and complicated cellular interactions are not interpretable without flow‐based dynamic simulation. In addition, the cell‐to‐cell junction, volume of luminal and abluminal liquids, and metabolic activities cannot be neglected as well. Thus, microfluidic technologies were applied in 3D BBB models, which precisely control both the cellular and extracellular microenvironment by mimicking cellular interactions and tissue structures, providing a strategy for stimulating the actual response of BBB, in a “organ‐on‐a‐chip” setting.^[^ ^199^ ^]^ Campisi et al. constructed a novel 3D BBB model replicating the microvascular network in fibrin gels using vasculogenesis strategies^[^ ^200^ ^]^ (Figure 11B). Peng et al. established a microfluidic‐based human blood–brain‐barrier (µBBB) that enables the co‐culture of endothelial cells, pericytes, and astrocytes simultaneously by manual coated µBBB chip channel using a covalently conjugated homogenous layer of extracellular matrix proteins.^[^ ^201^ ^]^ Another micro‐engineered BBB platform developed by Ahn et al. was further modified with reduced reactive astrogliosis stimulating the neuroinflammation under pathological conditions, which captured 3D NP penetration and distributions at cellular levels^[^ ^202^ ^]^ (Figure 11C). However, the previous models of NP delivery across BBB are mainly based on the traditional models with astrocytes and brain endothelial cells co‐cultured in transwell settings. Currently, several commercially available BBB models have been developed, such as the microfluidic device inverted by AIM Biotech Pte. Ltd. (https://aimbiotech.com/) and micro‐engineered physiological system‐tissue barrier chip (MEPS‐TBC) designed by Mepsgen Co., Ltd. (https://www.mepsgen.com/eng/pipeline/meps.php). The former involves co‐cultured human‐induced pluripotent stem cell‐derived endothelial cells (iPSC‐EC), brain pericytes, and astrocytes.^[^ ^199^, ^203^ ^]^ This latter MEPS‐TBC model is composed of endothelial cells lining blood vessels under blood flow, pericytes wrapping around the vessel, and astrocyte end feet in contact with the blood vessel.^[^ ^204^ ^]^ These innovative platforms addressing the complexity of physiological environment and dynamic simulation are highly recommended in future studies, which may offer alternatives for investigating BBB permeability of pharmaceuticals and NDC, and further enable a better understanding of the NPs delivery mechanisms involved in BBB.

### 6.3 Air–Liquid Interface Cell Exposure System Mimicking Inhaled NP Delivery

**Figure 12:**
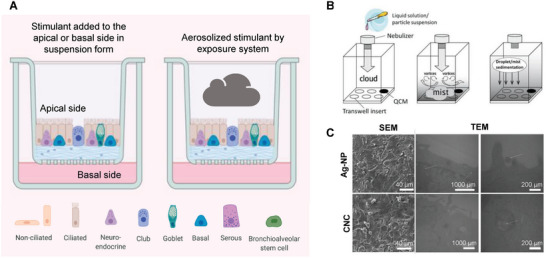
Air‐liquid interface cell exposure system mimicking inhaled NP delivery. A) Schematic illustration of the compound system of air‐liquid interface (ALI) culturing, with epithelial cells, macrophages, and fibroblast consist of the fundamental microenvironment in lung and bronchus, which addresses cell‐cell and cell‐stimulant interaction of inhaled particles. Reprinted with permission,^[^ ^208a^ ^]^ Copyright 2021, Elsevier. B) Schematic illustration of the ALI‐CLOUD device and experimental procedure. The nebulizer on top emits NPs droplet as cloud in the chamber, followed by deceleration and uniformity of the cloud by air drag, with the sedimentation of NPs mist exposed to the cell culture plate on the bottom. Reprinted with permission,^[^ ^211b^ ^]^ Copyright 2020, Springer Nature. C) Intracellular localization of ALI‐CLOUD exposed Au (upper) and gold cellulose nanocrystals (CNC, lower) NPs in A549 cells, which was analyzed with scanning electron microscopy (SEM) and transmission electron microscopy (TEM). Reprinted with permission.^[^ ^211a^ ^]^ Copyright 2023, Wiley‐VCH.

Air–liquid interface (ALI) is a realistic and efficient tool for mimicking inhaled particle delivery pathways, which assesses cell‐NP interaction and lung toxicity. With cells seeded on the apical compartment of the transwell inserts resembling the structure of an alveolar barrier, the ALI system is superior to conventional submerged cell culturing methods, which addresses cell‐cell and cell‐stimulant interaction of inhaled particles.^[^ ^205^ ^]^ Given that the epithelial cells, macrophages, and fibroblast consist of the fundamental microenvironment in the lung and bronchus, cell lines of these cell types were mono‐cultured or co‐cultured for developing the cell line‐based ALI system. For example, the Calu‐3 cell line with epithelial morphology, tight junction between cells, and secretory activity are used for lung drug absorption studies^[^ ^206^ ^]^ (**Figure** **12**A). BEAS‐2B cell line is valuable for studies investigating basic airway structure and functions.^[^ ^207^ ^]^ With similar characteristics of alveolar Type II pneumocytes in human lungs, A549 cell lines are one of the most used cell lines in toxicity studies.^[^ ^208^ ^]^ Despite the extensive utilization of cell line‐based ALI systems due to the easy access and guaranteed quality of commercial cell lines,^[^ ^209^ ^]^ the use of primary cells isolated from fresh tissues was considered as a better option at ALI conditions, which represents a heterogeneous population of different cell types. The ALI‐cultured primary human bronchial epithelial cells (HBECs) are a gold‐standard for human respiratory airway models.^[^ ^208a^ ^]^ However, the limited availability of primary cells is a great challenge for promoted utilization of primary cell‐based ALI systems, which encourages the development of commercial ALI models such as EpiAirway, OncoCilAir, and MucilAir.^[^ ^210^ ^]^ These models can be used in biomedical experiments with a long lifetime (3‐12 months), while the application is relatively inflexible without a customized design for specific research purposes.^[^ ^205^ ^]^

Using the ALI system, drugs or particles could be added to the apical side or basal side of the insert for conducting stimulant exposure. Novel ALI‐based approaches have been equipped with commercial gaseous exposure systems to test the cellular effect of gases in the gaseous phase for inhaled NPs and drugs, which allows precise exposing dosimetry without any interfering medium.^[^ ^211^ ^]^ (Figure 12B,C). More importantly, gaseous exposure in ALI‐based displayed similar therapeutic efficacy but faster uptake kinetics compared with submerged exposure,^[^ ^211b^ ^]^ which may pave the way for mimicking inhaled nanomaterials with a potentially more predictive cellular response.

### 6.4 Organ‐On‐A‐Chip Model

Organ‐on‐a‐chip, also known as tissue chips and micro‐physiological systems, are promising cell culture devices. Differing from the conventional cell culture methods, organ‐on‐a‐chip mimics the complexities of structures, microenvironment, and physiological systems in tissue and even organ units as a substitute for in vivo models, which offers a platform to study the biological functions and biomechanisms in vitro experiments.^[^ ^212^ ^]^ As for microengineering technologies, especially in the field of engineered NPs, these tissue chips have contributed to the investigations into the cellular or tissue targeted delivery,^[^ ^213^ ^]^ the therapeutic effect of drugs with NDC,^[^ ^214^ ^]^ and the potential adverse effects of NPs.^[^ ^215^ ^]^

**Figure 13:**
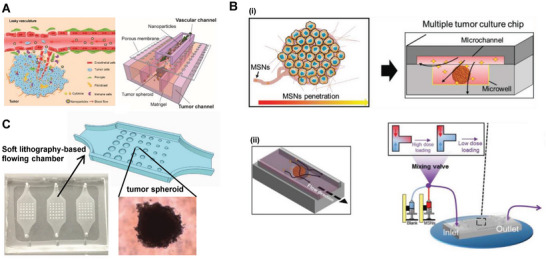
Organ‐on‐a‐chip model. A) Diagram illustrates the tumor microenvironment, which includes leaky vasculature and tumor tissues. It also presents a schematic of the TVOC model used in vitro. The TVOC model consists of a double‐layer device with two layers of microchannels separated by a porous membrane. The upper channel is designed for cultivating 3D tumor vasculature, while the lower channel replicates tumor tissue by incorporating tumor spheroids and a surrounding gel matrix. Reprinted with permission,^[^ ^217^ ^]^ Copyright 2018, ACS. B) The Multiple Tumor Culture Chip (MTC‐chip) is designed for assessing cellular MSN uptake. It includes a schematic diagram outlining design principles (i) and a comparison of different MSN administration routes on the chip, featuring a three‐way mixing valve inlet (ii). Reprinted with permission,^[^ ^218^ ^]^ Copyright 2019, Wiley‐VCH. C) Schematic illustration of a novel soft lithography‐based tumor‐on‐a‐chip, with an orderly matrix array of tumor spheroid cultured in hemispherical wells. Reprinted with permission,^[^ ^222^ ^]^ Copyright 2019, Wiley‐VCH.

The microfluidic‐based multilayered device using elastomeric materials‐based soft lithography, such as assembled poly(dimethylsiloxane) (PDMS) chambers, is one of the most common strategies. As an appropriate source for the fabrication of organ‐on‐chips devices, PDMS stands out from other materials with optical biocompatibility, and favorable oxygen permeability, which are advantageous properties for cell culture.^[^ ^216^ ^]^ Besides, its high transmittance allows imaging operations during the experiments, which could provide visualized evidence for research into the delivery path of NDC. For instance, Kwak et al. have proposed a tumor‐chip model mimicking the microenvironment constructed by tumor tissue, lymphatics, and blood vessels in breast cancer.^[^ ^213^ ^]^ In this model, microvascular endothelial cells and MCF‐7 cells were cultured on the upper and lower chambers of a PDMS‐based multilayer device, respectively, together with two additional channels simulating the adjacent lymphatics embracing the tumor mass. Based on this 3D model, the researchers investigated the delivery and elimination of fluorescent NPs in different diameters and provided evidence for the potential utilization of NPs in medical imaging technologies. A TVOC model features a two‐layer structure that encloses a porous polydimethylsiloxane (PDMS) membrane with 10 µm diameter pores. The purpose is to faithfully replicate the key characteristics of the tumor microenvironment^[^ ^217^ ^]^ (**Figure** **13**A). Additionally, tumor‐on‐a‐chip (TOC) platforms consider 3D tumor scaffolds and fluidic shear stress. For instance, Zhuang and colleagues developed the Multiple Tumor‐Culture Chip (MTC‐chip). This chip integrates 3D tumor spheroids, extracellular matrix (ECM), and dynamic administration into a single system. It serves to evaluate the cellular uptake and penetration depth of MSNs^[^ ^218^ ^]^ (Figure 13B). Neto et al. has introduced a microfluidic‐based neuro‐vascularized bone chip, mimicking the mechanistic interactions between innervation and angiogenesis in the inflammatory bone niche, which was validated by delivering anti‐inflammatory drug‐loaded NPs to counteract the neuronal growth associated with pain perception.^[^ ^219^ ^]^

In addition to the NP delivery research, modern organ‐on‐chips models are alternatives mimicking disease states instead of physiological situations to investigate the therapeutic efficiency and adverse effects of drug‐loaded NPs. Huh et al. described a biomimetic microsystem that reconstitutes the critical functional alveolar‐capillary interface of the human lung. This device revealed that cyclic mechanical strain accentuates toxic and inflammatory responses of the lung to silica NPs.^[^ ^215^ ^]^ In another study, the doxorubicin‐loaded hyaluronic acid NPs were exposed to breast cancer cells cultured in the multilayer device mentioned above.^[^ ^214^ ^]^ However, PDMS are likely to absorb hydrophobic molecules,^[^ ^220^ ^]^ suggesting an inaccurate drug response, and thus the results relevant to drug efficiency, toxicity, and cellular secretion are to be interpreted with caution.^[^ ^221^ ^]^ The chamber developed by Ran et al. has solved this problem, with an orderly matrix array of tumor spheroid cultured in hemispherical wells (Figure 13C).^[^ ^222^ ^]^ The NPs were delivered to the cells with microfluidics at specified flow rates, avoiding the toxic effects due to NPs sedimentation in conventional culturing methods. Similarly, compared with mono‐cultured models, there is an improved survival rate of cancer cells in multilayer devices,^[^ ^212^ ^]^ which is partially because of the endothelium barriers for NPs transportation, together with the fluid flows reducing the attachment of NPs.

Despite the considerable progress, the organ‐on‐chips are yet remaining an imperfect alternative to in vivo *models*. The results generated from organ‐on‐chips models are highly recommended to correlate with those from in vivo experiments, or even with human clinical data for validation. Besides, the immune response of organs to exogenous stimulations cannot be measured. With the novel lymph nodes‐on‐a‐chip, the current organ‐on‐chips are exhorted with great potential to construct an integrated complex of both organ and immune system.^[^ ^223^ ^]^ Recently, induced pluripotent stem cells (iPSCs) have been applied in novel organ‐on‐chips as a promising cell source,^[^ ^224^ ^]^ which may enable more human‐related analysis and even predict physiological responses for patient‐specific disease.^[^ ^212^, ^225^ ^]^ Besides, current models are designed to mimicking the complex functioning units which are required to address specific applications for nanomedicine evaluations. Despite the limited studies utilizing vascular‐based platforms, lymphatic systems are often integrated into comprehensive organ‐on‐chip models and established immune‐related models have not yet been used as a platform for the study of nanomedicine. Future organ‐on‐chip devices are of high importance to be developed with multiple systems such as advanced vascular,^[^ ^219^ ^]^ lymphatic,^[^ ^226^ ^]^ and immune system devices,^[^ ^227^ ^]^ which are about to simulate both the structure and function of the physiological systems, contributing to further research in nanomedicine.

### 6.5 Organotypic Model Precision‐Cut Tissue Slices

**Figure 14:**
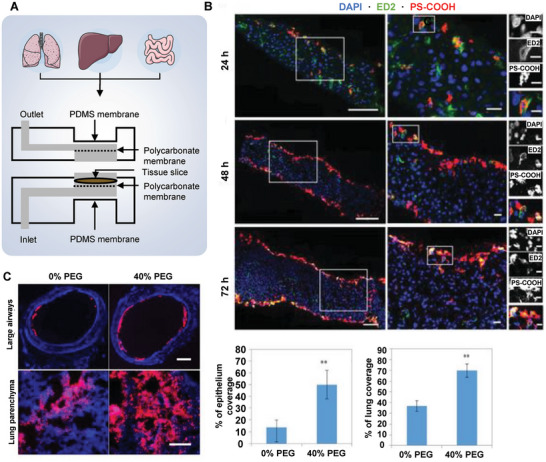
Organotypic model with precision‐cut tissue slices for studying nano‐bio interactions. A) The precision‐cut tissue slices (PCTS) are cut from different organs with a diameter of 5 mm and thickness of ≈250–500 µm, containing about 10–20 cell layers, covering all cells presented in tissue, and cultured ex vivo. B) Cross‐sections of liver slices exposed to far‐red PS‐COOH NPs at a 10 µg mL^−1^ concentration for 24, 48, and 72 h were obtained using confocal fluorescence microscopy. Scale bars (from the left): 100, 40 and 10 µm. Blue: DAPI‐stained nuclei. Red: PS‐COOH nanoparticles. Green: ED2+ Kupffer cells. Reprinted with permission,^[^ ^231b^ ^]^ Copyright 2020, Informa UK. C) Representative images depicting the distribution of NPs (in red) in the major airway epithelium and lung parenchyma following the administration of the respective NPs. Blue: DAPI‐stained nuclei. The scale bar: 0.2 mm. Image‐based quantification of NP coverage within the major airways and the distribution of NPs within the lung parenchyma. Reprinted with permission,^[^ ^232a^ ^]^ Copyright 2018, Elsevier. B.V.

The precision‐cut tissue slices (PCTS) are generated from different organs in healthy or diseased conditions and could be cultured over a few days to weeks even months ex vivo.^[^ ^228^ ^]^ With a diameter of 0.5–2 cm and thickness of ≈250–500 µm, one tissue slice contains about 10–50 cell layers, covering all cell types present in the tissue, and presenting the complex architecture of tissue with real cellular abundance and interactions^[^ ^229^ ^]^ (**Figure** **14**A). Compared with cell‐culture strategies and in vivo animal experiments, this organotypic model constitutes a valuable alternative with vivid mimicking of sophisticated physiological or pathological features, which is also a time‐ and cost‐saving approach for nano‐engineering studies.^[^ ^230^ ^]^

To date, researchers have primarily utilized PCTS to investigate NP induced inflammation and toxicity, with a predominant focus on organs such as the liver,^[^ ^231^ ^]^ lung,^[^ ^232^ ^]^ intestine,^[^ ^233^ ^]^ and tumor tissues^[^ ^234^ ^]^ using both animal and human samples. While these studies have provided insights into NP uptake, they often lack detailed information regarding the specific cell types involved, quantitative analyses of NP distribution, and the underlying mechanisms of NP‐cell interactions. For example, Bartucci et al. employed 3D ex vivo mouse PCTS models to examine the uptake and effects of silica, carboxylated, and amino‐modified polystyrene NPs in organs like the liver, lung, and kidneys. This research revealed notable variations in NP distribution among different organs, with minimal uptake observed in kidneys, increased accumulation in lung slices and macrophages.^[^ ^232b^ ^]^ In the liver, exposure to carboxyl‐modified polystyrene NPs resulted in the accumulation of a substantial number of NPs within Kupfer cells. Intriguingly, these NP‐laden cells exhibited a tendency to migrate within the tissue towards the section boundaries^[^ ^231b^ ^]^ (Figure 14B). Similarly, another study explored the distribution of 40% PEGylated NPs in mouse lung PCTS, highlighting a broad and more uniform dispersion compared to the inhalation of non‐PEGylated NPs^[^ ^232a^ ^]^ (Figure 14C). However, it's important to exercise caution when interpreting the outcomes of PCTS‐based particle‐tissue interaction models, especially when deducing cellular responses and underlying mechanisms. These models may not fully replicate the complexity of NP‐tissue interactions in vivo due to the submerged tissue culturing conditions, where all cell types are equally exposed to dispersed NPs.^[^ ^231b^ ^]^ Nevertheless, a recent single cell RNA sequencing study revealed multiple in vivo cellular circuits such as the epithelial, mesenchymal, and endothelial cell transitions during lung fibrogenesis can be induced in ex vivo human lung PCTS,^[^ ^235^ ^]^ highlighting the huge potentials of PCTS in tissue perturbations, drug screening and toxicology test. Researchers should therefore consider targeted delivery strategies for NDCs to specific tissue regions or cell types within PCTS, as this step is crucial for advancing the application of NDCs in preclinical and clinical studies.

## 7 Imaging‐Based NP‐Tracing Techniques in Intracellular Delivery

Detecting individual or small clusters of NPs within single cells, tissues, or organisms poses a significant challenge, particularly given that only a minority of delivered NPs can effectively reach tumors in vivo.^[^ ^236^ ^]^ Current in vivo non‐invasive evaluation of the delivery, distribution, and dosage of NDCs largely depends on the imaging‐based techniques, for instance, the imaging biomarkers of cancer nanomedicine utilizing of MRI, PET, and CT are likely required for patient stratification.^[^ ^237^ ^]^ Photoacoustic imaging and whole‐body fluorescence or bioluminescence imaging (IVIS) are also commonly used for in vivo visualization of NDC distribution, despite lacking the ability to observe single or small clusters of NPs at cellular resolution.^[^ ^123^, ^238^ ^]^ By modulating the percentage of DOTAP, specific accumulation of LNP‐encapsulated Cas9/sgRNA complexes can be clearly visualized in multiple organs, including muscle, brain, liver, and lungs using the in vivo imaging system.^[^ ^238a^ ^]^ This NDC allows for multiplexed and tissue specific gene editing in mice. Despite the (semi‐)quantitative information of NDC dosage can be provided with those macroscale imaging techniques mentioned above, it is still not sufficient to determine the cell‐specific targeting in NDC delivery science. We thus established a new methodology named tissue clearing and light sheet fluorescence imaging (LSFM), which enables quantitative mapping NP distribution and dosimetry within cellular resolution.^[^ ^239^ ^]^ Recently, we further developed an artificial intelligence powered 3D imaging pipeline, which permits automatic segmentation of the entire lung bronchial airway structure, providing in‐depth, cellular‐resolution 3D profiling of NP dose within the bronchial or alveolar region of the lung.^[^ ^240^ ^]^ This technology is of critical significance as it bridges the gap between noninvasive in vivo imaging and the destructive ex vivo 2D imaging of histological slices.

There is a growing call for NDC studies to prioritize a deeper comprehension of drug delivery and fundamental biology when applied to theranostic applications, due to the limited availability of NDC formulations in clinical settings. The term “Seeing is Believing” thus holds paramount significance in the nanomedicine application domain. For example, by utilization of a couple of high‐resolution imaging techniques (TEM, optical imaging such as confocal microscopy and LSFM), Chan group recently demonstrated that NPs transportation occurs through the trans‐endothelial pathway^[^ ^9^ ^]^ and NPs leaving tumors via lymphatics re‐enter the bloodstream, recirculating to engage the tumor again,^[^ ^10^ ^]^ challenging the conventional EPR theory. Indeed, the majority of NDC application studies, including NP characterization and NP‐cellular interactions, rely on techniques such as electron microscopy and optical microscopy, as illustrated in all Figures presented here. An ideal imaging method should provide sufficient resolution, quantitative data, simplicity in sample preparation, and high throughput. Optical microscopy enables qualitative and quantitative analysis with subcellular spatial information in vitro and ex vivo. Recent advances on super‐resolution fluorescence microscopy techniques like structured illumination microscopy^[^ ^241^ ^]^ and stimulated emission depletion microscopy^[^ ^242^ ^]^ offer even higher spatial resolution (10‐100 nm), allowing for the visualization of single NDC within cells. However, fluorescence‐based methods face challenges related to specific labeling, stability, fluorophore degradation, and potential impacts on cellular responses.^[^ ^243^ ^]^ Considerations must also be given to issues like NP aggregation, uneven fluorophore distribution, and limited detector sensitivity. Indeed, this review provides an overview of the advantages and limitations of various novel fluorescent material‐based strategies and rapidly advancing imaging methods (**Table** **2**) for tracking NDC interactions at single‐cell resolution across diverse biological contexts.

**Table 2:** Characteristics of the advanced microscopy techniques.

	Advantages	Disadvantages	Maximum magnification	Resolution	Reference
CLSM	high‐resolution vertical imaging and 3D imaging non‐destructive user‐friendly	slow imaging destructive to live cells due to high‐intensity laser illumination not suitable for colloidal system	100 x	140 nm (lateral) and 1 µm (axial)	[331]
SEM	visualization of high magnification	only surface characterization	100,000 x	2‐10 nm	[332]
	identification at molecular level	complicated sample preparation			
	applicable to both cells and tissues	vacuum environment needed			
		low resolution at nanoscale			
TEM	high resolution and magnification	overlapped images	1,000,000 x	<1 nm	[273, 275, 333]
	morphology and size distribution of NPs	complicated sample preparation			
	identification at molecular level	vacuum environment needed			
		not suitable for 3D visualization			
AFM	high resolution and magnification	slow imaging	depend on multifactors	0.5 nm	[290, 334]
	applicable for living cell samples	effect of samples by probe tips			
	3D imaging				
	identification at molecular level				
	user‐friendly				
Raman	no sample preparation process	slow imaging	NA	1 µm	[335]
	3D imaging	multiple factors affecting the accuracy			
	user‐friendly	relatively low resolution			
LSFM	3D volumatic imaging Rapid imaging with cellular resolution Large size datasets	Require optical tissue clearing Tissue volumes might be changed Reagents might be toxic Photobleaching of NDC signals	20X	400‐500 nm	[239, 336]

### 7.1 Aggregation‐Induced Emission (AIE) Techniques

**Figure 15:**
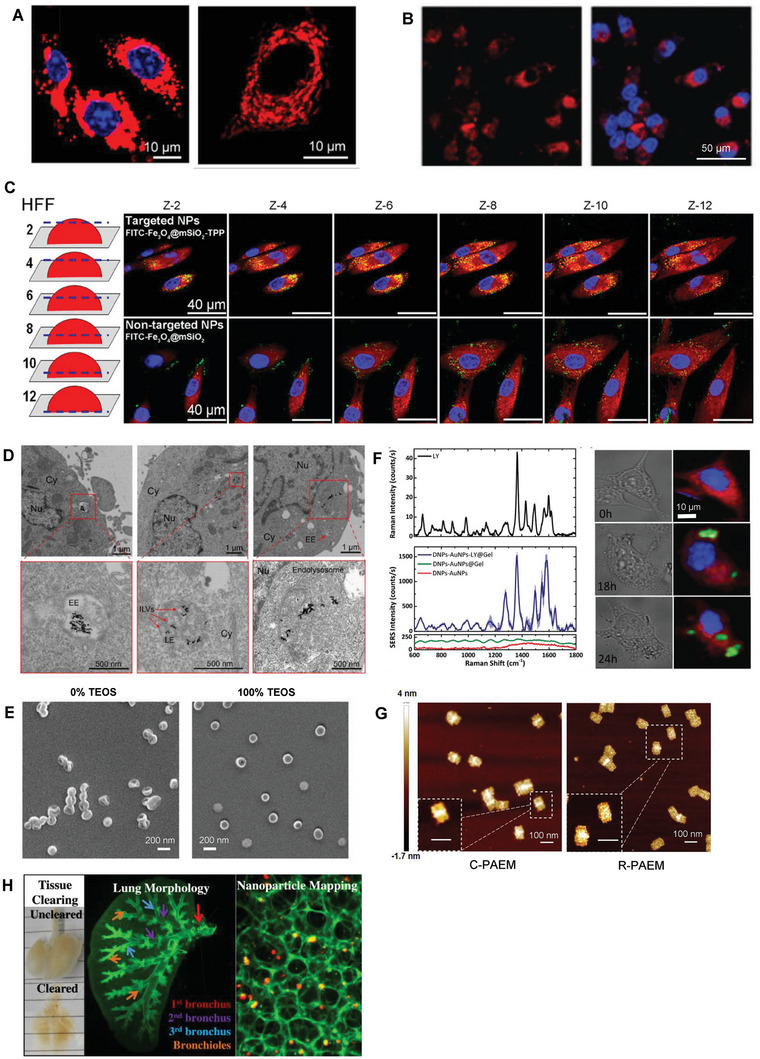
Imaging‐based NP‐tracing techniques in intracellular deliveries. A) Representative confocal images of cytoplasm and mitochondria of MCF‐7 cancer cells labeled by AIE dots. Reprinted with permission,^[^ ^245^ ^]^ Copyright 2018, American Chemical Society. B) Representative confocal laser scanning images showing the internalization of a novel personalized upconversion tumor‐pH‐sensitive photodynamic nanoagents (PPNs, red signals) into A549 cells (blue signals) under mild acid environment (pH 6.5). Reprinted with permission,^[^ ^309^ ^]^ Copyright 2018, Wiley‐VCH. C) Schematic illustration of Z‐stacked imaging sequence by confocal laser scanning microscopy (CLSM, left), and representative CLSM images of the particle internalization into HFF cells. Reprinted with permission,^[^ ^310^ ^]^ Copyright 2015, American Chemical Society. D) Representative TEM images show the subcellular localizations of internalized AuNPs in the early endocytic structures (at 1 h), in the multivesicular bodies (at 3 h), and in the endolysosomes (at 24 h). Reprinted with permission,^[^ ^311^ ^]^ Copyright 2021, American Chemical Society. E) SEM micrographs of silica nanocapsules synthesized using 0% (softest) and 100% (stiffest) of tetraethoxysilane (TEOS). Scale bars: 200 nm. Reprinted with permission,^[^ ^270^ ^]^ Copyright 2020, AAAS. F) Representative Raman spectrum (left) of Galunisertib (LY2157299, LY, black line) and LY‐loaded golden diatomite NPs (DNP‐AuNPs, blue line), together with the background signals gelatin capped DNP‐AuNPs (DNP‐AuNPs@Gel, red line) and the DNP‐AuNPs alone (green line). Sequential optical image and Raman mapping images after co‐incubation of colorectal cancer cells and DNP‐AuNPs‐LY@Gel, showing the internalization of NPs into the tumor cells (right). Reprinted with permission,^[^ ^312^ ^]^ Copyright 2021, Wiley‐VCH. G) Different densities of positively charged polymer patterns on DNA‐origami structures (low density: C‐PAEM and high density: R‐PAEM) were formulated to investigate the surface charge effects on cellular uptake of particles. Reprinted with permission,^[^ ^295^ ^]^ Copyright 2022, American Chemical Society. H) Tissue cleared light sheet fluorescence microscopy enables 3D co‐mapping of tissue morphology (tissue autofluorescence in green: the airway and alveoli) and inhaled NP distribution (red) within cellular resolution in intact lungs. Reprinted with permission,^[^ ^239^ ^]^ Copyright 2019, American Chemical Society.

In contrast to advanced microscopy techniques, aggregation‐induced emission (AIE) strategies have concentrated on improving the fluorescence intensity of the NDC as well as the cells to acquire high‐quality images. Fluorogens with AIE feature (AIEgens) are non‐emissive with weak fluorescence in a molecular state, but an aggregation of the AIEgens can induce intensive emission as the result of intramolecular motion restriction.^[^ ^244^ ^]^ AIEgens encapsulated NPs have been observed to have higher fluorescence intensity and better biocompatibility, compared with those loaded by inorganic fluorophores^[^ ^245^ ^]^ (**Figure** **15**A). To encapsulate AIEgens into NPs, strategies have been developed in various studies, with two commonly applied methods in various applications. The first utilized hydrophilic polymers or biomolecules to conduct chemical modification of AIEgens, which achieves amphiphilic properties of AIEgens, and results in self‐assembling into NPs.^[^ ^246^ ^]^ The drawbacks of this strategy are obvious with the altered size distribution of NPs and the time‐consuming modification process. The second approach was developed to solve these problems, with novel AIEgens encapsulated particles generated by nanoprecipitation methods.^[^ ^245^ ^]^ Consequently, these NPs have been intensively used with better water solubility and stronger emission with anti‐photobleaching fluorophores. Besides, recent studies have also tried other strategies to produce amphiphilic AIEgens, which have provided the possibility for large‐scale production and any alterations to NP size distributions.^[^ ^247^ ^]^

Meanwhile, advanced AIE techniques have also been developed for targeted organelle imaging. With functional units, AIEgens are designed to specifically interact with cytoplasm membrane,^[^ ^248^ ^]^ lysosome,^[^ ^249^ ^]^ mitochondria,^[^ ^250^ ^]^ nucleus,^[^ ^251^ ^]^ and so on, which brings sub‐cellular information and provides access to explore the fate of NDC after the transmembrane displacement. Despite the relatively rapid developing progress of the AIE techniques in imaging NP‐cell interaction, there are also challenges to be considered. Current research has been focused on the improved biocompatibility of AIE‐encapsulated NPs for their better application in disease diagnosis and treatment, which might affect the actual response of the cells.^[^ ^252^ ^]^ Besides, the nonspecific toxicity of AIEgens to cells cannot be ignored even at a very low level.^[^ ^253^ ^]^ Without a standardized evaluation platform and well‐controlled study design, the NP internalization efficiency and cellular responses are not perfectly accurate under current AIE strategies.

### 7.2 Upconversion NPs (UCNPs)

Similar to AIE techniques, the upconversion NPs (UCNPs) also focus on particle modification to achieve super‐resolution imaging. Lanthanides ions were fixed to the material as sensitizers and activators, which can absorb near‐infrared (NIR) photons and accept transferred energy respectively during the upconversion progress, leading to the visible photon to realize luminescence and imaging^[^ ^254^ ^]^ (Figure 15B). The evident advantages of UCNPs are the non‐photobleaching and non‐photoblinking properties, which make up for the deficiency of conventional organic dyes and avoid poor‐quality images which might result from the photobleached fluorophores.^[^ ^255^ ^]^ Besides, UCNPs barely introduce autofluorescence background due to the inactive response of the biological sample to NIR light,^[^ ^256^ ^]^ which allows clearer images and wide biomedical applications.^[^ ^257^ ^]^ However, the flexibility to encapsulate lanthanide ions into specific NDC is limited,^[^ ^258^ ^]^ which narrowed the application of UCNPs. The alteration in NPs physicochemical properties introduced by lanthanides should also be considered. A recent study highlighted the development of avalanching NPs (ANPs) that can be photoswitched bidirectionally using near‐infrared (NIR) light. These ANPs enable full optical control of upconverted emission and can be photoswitched indefinitely (more than 1,000 cycles over 7 hours) without measurable photodegradation.^[^ ^259^ ^]^

### 7.3 High (Super)‐Resolution Fluorescence Microscopy

To improve the resolution of fluorescence microscopy, advanced techniques have been developed including confocal laser scanning microscopy (CLSM), photo‐activated localization microscopy (PALM), and stochastic optical reconstruction microscopy (STORM) to achieve precise localization of the NDC within cells/tissues. Among these instruments, CLSM utilized a relatively narrow field depth and the point scanning technique to exclude the disruptive objects which are out‐of‐focus, obtaining images with improved lateral resolution as well as serial optical sections with high z‐axial resolution. Earlier research utilizing CLSM mainly focused on the effects of NP physicochemical properties, including morphology, size, dispersion and aggregation, and bioactive stability on NP‐cellular interaction (Figure 15C). Besides, similar to conventional fluorescence microscopy, the fluorescence intensity obtained in CLSM images also provides access to conduct a sub‐quantitative evaluation of the delivery efficiency compared between different exposure doses or time points.^[^ ^260^ ^]^ Different from the conventional dyed NPs loaded with fluorophores,^[^ ^261^ ^]^ advanced materials such as NP‐antibody conjugates^[^ ^262^ ^]^ and NP functionalization^[^ ^263^ ^]^ have offered new solutions to specific NDC. However, the power of laser illumination may harm physiological cellular behavior, and there is an inevitable pretreatment process for fixed specimens, both of which should be carefully considered in the interpretation of living‐cell imaging regarding cell‐NP interactions.^[^ ^264^ ^]^ A one‐photon excitation fluorescence imaging window in the 1700–2000 nm (NIR‐IIc) range was recently achieved using biocompatible core‐shell lead sulfide/cadmium sulfide quantum dots and superconducting nanowire single‐photon detectors. This technology allows for non‐invasive, high‐resolution imaging through live mammal tissues, including cellular‐resolution imaging of small blood vessels and immune cells in the mouse head and lymph nodes without surgery.^[^ ^265^ ^]^

Meanwhile, PALM and STORM have improved the spatiotemporal resolution and quality of the images, which achieves dynamic location of the fluorescent point source by either photobleaching or on/off state switching, respectively.^[^ ^266^ ^]^ Despite the common utilization and substantial improvement of STORM for investigation of the cell‐NP interaction and intracellular NP trafficking, there are still limitations. For instance, the STORM system relies on the fluorophores in the buffer mixture, which are excited by different light sources. With these fluorophores, the medium is non‐physiological, toxic, and inadequate to live‐cells, which limited the utilization of STORM in real‐time monitoring of living cells.^[^ ^267^ ^]^ Besides, the STORM imaging system requires two light sources,^[^ ^268^ ^]^ which brings additional burden. Erstling et al. encapsulated the fluorophores using aluminosilicate NPs, which achieved the non‐toxic environment and applicable dye blinking with a single excitation source due to the photoinduced redox process of the aluminum, allowing the visualization of the NP internalization in living cells.^[^ ^269^ ^]^ Furthermore, the combination of the functionalized fluorophores and antibodies realized the targeted imaging in the STORM system, as well as quantitative measurements of the particle numbers at the subcellular level.^[^ ^269^ ^]^

### 7.4 Electron Microscopy

Microscopic physical and chemical structure characteristics determine the macroscopic properties of particles, and it is of great significance to carry out an accurate and detailed analysis. Applied from structural biology to nanoscience, electron microscopy (EM) has been utilized as an indispensable tool in investigations into cell‐NP interactions, including scanning electron microscopy (SEM) and transmission electron microscopy (TEM)^[^ ^243^, ^270^ ^]^ (Figure 15D,E). Based on the basic information obtained by electron microscopies such as atomic periodicity, elemental composition, and electron density, objective observation was achieved for the physical morphology of NPs, including particle size, surface roughness, dispersion and aggregation status, and microchemical composition of nanomaterials.^[^ ^271^ ^]^ Besides, EM is the only method available for the visualization of single non‐tagged NPs in cells.

Superior to photon‐based strategies, TEM possesses a resolution of ≈0.2 nm or higher^[^ ^272^ ^]^ and collects both structural and chemical composition properties of NPs with sizes of 1–100 nm according to its interaction with a collimated exposed electron beam with small de Broglie wavelengths.^[^ ^271^ ^]^ Electro beams (80‐300 kV) produced by thermionic sources and field‐emission sources transmit through the NP samples dispersed in the liquid substrate and interacting with its internal structures.^[^ ^271^ ^]^ Generated beams were accelerated by anode of 40–400 kV and then focused by electromagnetic and electrostatic lenses for the final irradiation on the samples.^[^ ^273^ ^]^ The electrons scattered or absorbed by the specimen are collected and magnified to provide information for the picture of samples, which could be displayed on the screen. As an imaging tool with relatively high resolution and valuable complement to conventional instruments for the measurement of particle sizes, TEM displays great advantages in the identification and visualization of the structure of nanomaterials, dispersion status of samples, orientation, and exfoliation of NP individuals. Besides, TEM also provides accurate information to confirm the success of encapsulation in the construction of complicate NP delivery systems.^[^ ^274^ ^]^

However, the complicated preparation process of specimens and high requirements of sample quality become the main obstacles to the further application of TEM. Due to the low contrast among biological structures, TEM remains inadequate for the visualization of living specimens.^[^ ^275^ ^]^ 1Although SEM requires an easier sample preparation process compared with TEM, the dehydration step is also unavoidable, which limits the utilization of SEM in dynamic monitoring of the cellular internalization of NPs. Thus, cryo‐TEM and freeze‐fracture imaging approaches have been introduced to provide 3D images and to investigate the interactions between NPs and living cells.^[^ ^276^ ^]^ Peckys and colleagues have introduced silicon nitride membranes into TEM, which allows the imaging of living cells inside the microfluidic chamber.^[^ ^277^ ^]^ However, since the cellular structures are invisible in this technology, optical or fluorescence microscopies are required in parallel experiments. Along with the information on cell numbers from the EM images to estimate an average value of the internalized NPs in each cell, which has improved the technology as a quantitative measurement.^[^ ^277^ ^]^ Alternatively, the sequential analysis of cross sections of cells also allowed 3D visual images of individual cells, providing clear and intuitive observation of the NPs distribution in cells.^[^ ^278^ ^]^

### 7.5 Raman Microspectroscopy

Unlike electron microscopy, Raman microspectroscopy is based on the elastic scattering of the photons and molecular vibrations caused by the interaction between monochromatic laser beam and samples, which produces a “fingerprint” spectrum of the molecular bond representing the material characteristics, allowing the identification of chemical composition as well as the microenvironment without fluorescent staining or labeling (Figure 15F). The studies have revealed that formalin is served as the best fixative, which preserves proteins and cellular integrity, producing the spectral content closest to that in living cells.^[^ ^279^ ^]^ The confocal mode of Raman spectroscopy utilized in 2D/3D cell culture environments also allows in vitro analysis of fixed and living cells at the subcellular level,^[^ ^280^ ^]^ which could provide information on the cellular trafficking of NDC. Besides, in combination with multivariate analyses such as K‐means clustering, biochemical information of nucleic acids, proteins, lipids, and carbohydrates are detectable, reflecting the metabolism alterations in cells exposed to NPs,^[^ ^281^ ^]^ which makes further studies into cellular response and internalization pathways possible.

Utilizing Raman microspectroscopy, visualization of NPs transmembrane transport into single cells has been achieved for magnetic NPs,^[^ ^282^ ^]^ metallacarborane aggregates,^[^ ^283^ ^]^ and carbon NPs.^[^ ^284^ ^]^ Dorney et al. have identified spectral cluster with features of polystyrene in A549 cells exposed to polystyrene NPs, as well as its cytoplasmic and perinuclear localization, which provide access to the investigation into the cellular internalization process of NPs.^[^ ^285^ ^]^ Besides, Moore et al. have explored the interaction between molybdenum disulfide (MoS2) and the macrophage‐like cell line THP‐1 in vitro^[^ ^286^ ^]^ using combined single‐cell mapping of Raman microspectroscopy and pulse‐chase approaches, which identified the different microenvironment during the intercellular trafficking process. Furthermore, the degradation patterns and spectral profile of biodegradable NPs are achievable using Raman microspectroscopy. However, most of the studies using Raman microspectroscopy were aimed at investigations into the mechanism of the transmembrane process and intracellular fate of particles, while those evaluating the uptake efficiency of NPs are yet limited.

One of the drawbacks of Raman microspectroscopy is the relatively weak signal and the concealed signal by fluorescence or stray light scattering from the background.^[^ ^287^ ^]^ Several methods have been proposed to improve the signal‐to‐noise ratio and signal intensity, including surface‐enhanced Raman scattering (SERS) utilizing noble metal surfaces^[^ ^288^ ^]^ or carotene coating^[^ ^284^ ^]^ for enhancing Raman scattering, and coherent Raman scattering for improving the readout speed as well as technique sensitivity,^[^ ^289^ ^]^ which are both widely used for visualization of intracellular NDC and single cell analysis.

### 7.6 Atomic Force Microscopy

Atomic force microscopy is a powerful instrument based on the electron method. Driven by piezoelectrics, the scanning cantilever is removable to measure the interaction between the scanning probe and sample with a lateral resolution of sub‐nanometers, which is comparable to EM.^[^ ^290^ ^]^ In addition, the AFM could be conducted in an aqueous circumference without staining, which allows a more flexible experimental environment compared with the vacuum states required by EM.^[^ ^291^ ^]^ The water also provides access to maintain the biological morphology and physiological response of cells when exposed to NDC. Despite its flexibility, the possibility of tip contamination and cellular membrane deformations introduced by the exact contact between the scanning probe and samples should be carefully considered as the main drawbacks of AFM. Due to its convenient operation and high efficiency of measurement, numerous studies have utilized AFM to characterize the morphological and physicochemical properties of NDC systems, including functional AuNPs,^[^ ^292^ ^]^ PLGA and RGD/PLGA‐based nanofibers,^[^ ^293^ ^]^ and PVA stabilized nanospheres,^[^ ^294^ ^]^ and polymer‐DNA origami nanostructures^[^ ^295^ ^]^ (Figure 15G). However, studies investigating the cellular uptake efficiency or NP‐cell interaction are yet limited.

### 7.7 Tissue‐Cleared Light Sheet Fluorescence Microscopy

Different from traditional whole body/organ fluorescent imaging (such as in vivo imaging system, PerkinElmer), tissue‐clearing LSFM offers a holistic view of intact organs or organisms within cellular resolution. The concept of optical tissue clearing is to match the refractive index of tissue with clearing reagents such as the dibenzyl ether (DBE) or a mixture of benzyl alcohol and benzyl benzoate (BABB) followed by dehydration and liquid remove, producing a transparent organ/organism that can be fully illuminated light slice‐by slice using LSFM.^[^ ^239^ ^]^ Recently, tissue‐clearing LSFM has been used to obtain a high‐resolution 3D view of NP accumulations in different tissues/organs. For example, intravenously administered QDs were observed in the transparent liver, spleen, and tumor of mice enabled by a hydrogel‐based clearing method (CLARITY,^[^ ^296^ ^]^ tissues treated with an acrylamide hydrogel and sodium dodecyl sulfate (SDS), and glycerol) and the distribution of QDs to the vessel were quantitatively computed and mapped.^[^ ^297^ ^]^ Light scattering of metal NPs in optically cleared tissues enhanced by in situ chemically grown approaches allows for supersensitive detection of low‐accumulation gold and silver NPs under dark‐filed microscopy.^[^ ^298^ ^]^ Combining the dark filed imaging of NP scattering signals with fluorescence‐labeled cancer cells and blood vessels in the optical‐cleared livers and lungs using LSFM, Kingston et al. developed a machine learning‐based image analysis to evaluate the penetration of NPs into the primary tumors and micrometastases with single‐cell resolution and found more than 50% of cells in micrometastases ingested NPs, while only 17% of cells in primary tumors contained NPs.^[^ ^299^ ^]^ Also, quantitative tissue‐cleared optical imaging of tumor‐associated macrophages (TAMs) using ^64^Cu‐labeled polyglucose NPs (Macrin) in an orthotopic and immunocompetent mouse model of lung carcinoma, Kim et al. demonstrated that Macrin was mainly taken by TAMs with striking heterogeneity of TAM counts in different tumor regions, where TAM‐rich tumors exhibited enhanced nanotherapeutics accumulation compared to TAM‐deficient tumors.^[^ ^300^ ^]^ More importantly, whole‐organ characterization of inhalation delivery and deposition has been achieved using tissue‐clearing 3D imaging, in which we showed that the ventilator‐assisted aerosol inhalation resulted in a deep and uniform acinar deposition of fluorescent NPs compared to the central, patchy deposited features by intratracheal instillation.^[^ ^239^ ^]^ Holistic features of NP biokinetics and cellular fate including macrophage mediated acinar migration of NPs in the entire lung after various routes of pulmonary delivery were clearly revealed by artificial intelligence assisted LSFM imaging.^[^ ^240^ ^]^ Multimodal imaging including X‐ray phase contrast imaging in vivo and ex vivo LSFM further permits simultaneous visualization of the real delivery dynamics of NPs through the trachea to deep lung and subtle localization of NPs in lung epithelium.^[^ ^237b^ ^]^

The current main drawbacks of tissue‐cleared LSFM imaging are the risk of degradation and photobleaching of NPs during the optical clearing process, tissue morphology or physiology alteration due to the tissue shrinkage/expansion and loss of cellular protein, difficulties in imaging lipid or polymeric NPs such as liposomes and poly (lactic‐co‐glycolic acid) (PLGA) that are currently used in clinics, and challenges in the processing of large‐sized imaging data. One of the possible solutions faced for lipid NPs is to seek more gentle aqueous‐based tissue‐clearing protocols such as Clear^T^, SWITCH, and MACS,^[^ ^296^, ^301^ ^]^ all of which possess ideal compatibility with multiple probes, especially for lipophilic dyes (*e.g*., DiD and DiI dyes) and liposomes. An alternative strategy is to develop peptide tags that could crosslink or stick to the tissue proteins and can be preserved during lipid removal, for example, liposomes conjugated with a peptide tag (REMNANT) and a Cy3 dye were detected in tumors, while it is not visible for lipid dye Dil after various kinds of tissue clearing including the CUBIC, CLARITY, and 3DISCO.^[^ ^302^ ^]^ The authors also found that peptide tag labeling had low/no effect on liposome distribution in vivo and the degradation of liposomes in vivo is more than 100 times faster than in vitro conditions. The other disadvantages are out of scope and are not discussed in detail here.

## 8 Conclusions and Future Perspectives

The utilization of NPs in nanomedicine for biomedical applications such as drug delivery, imaging, and disease diagnosis and therapy is exponentially increased over the past decades. Researchers have committed to develop new NDC formulations, novel delivery technologies, and advanced treatment regimens to maximize drug efficacy and minimize side effects. Efficient delivery of NDC to single cells, injured tissue sites, and diseased organs (including solid tumors) requires a comprehensive understanding of the active or passive transport pathways across multiple biological barriers and subsequent nano‐biological (nano‐bio) interactions. Until now, the active endocytic process has been well‐documented that smaller NPs are predominantly ingested by cells via clathrin‐ and caveolae‐mediated endocytosis, while macropinocytosis and phagocytosis are responsible for uptake of bigger NPs and their agglomerates. The exact mechanisms of cellular internalization for many novel‐designed NDC, however, are not yet fully understood, and has to be studied individually. Despite increased knowledge of nano‐bio interactions, multiple transport pathways including the unexplored ones might be involved in the NP internalization, reflecting the complexity during NP delivery.^[^ ^12c^ ^]^ Passive NP delivery via membrane‐disruption technologies thus provides well‐controlled and high‐efficient capabilities for precision intracellular NDC delivery. The inconsistency of results can still be observed in the literature due to multiple factors e.g., variations in NDC types, various cell types and organisms, different experimental conditions, as well as cell heterogeneities like drug‐resistant versus drug‐responsive cells. When studying the delivery capability of new NDC in vitro, it is of critical relevance to take full consideration of favorable cell types, NP characteristics, and delivery methods.

**Figure 16:**
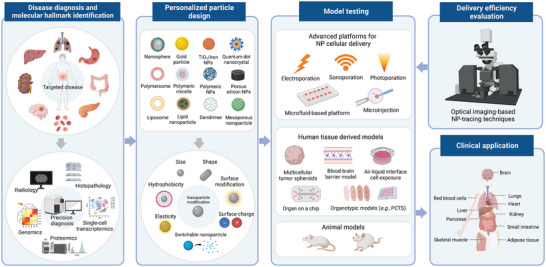
A comprehensive personalized NP delivery framework for clinical translation. It involves disease diagnosis and biomarker identification using histopathology and multi‐omics technology, designing novel NDC considering NP types and properties, selecting effective delivery platforms, utilizing emerging pathophysiological models derived from human tissues, as well as employing new analytical tools like microscopic NP‐tracing techniques for systemically understanding the NDC delivery efficiency, nano‐bio interaction, and diagnostic/therapeutic outcome.

NDC systems provide an assortment of versatile features like particle size, shape, charge, hydrophobicity, elasticity, and surface functionalization that can be tailored to optimize delivery for individual applications and personalized therapy. Those NP features influencing the targeting efficiency and nano‐bio interaction across various biological conditions were reviewed here to provide a reference for future NDC design and clinical translation. However, customized NP formations might only be applicable to very specific conditions, cell lines, or model organisms, limiting the translation of relevant findings to broad situations. Also, a large number of non‐commercial NDC applied in different laboratories probably were not prepared or formulated under a strict and standardized protocol, which, in worse cases, could potentially result in the unknown identity of NDC. The subsequent biological effects associated with those NDC could thus be attributed to other factors other than the NP itself, such as by‐products during NDC synthesis, chemical dispersants (for NP solubility), and contaminations.^[^ ^2^ ^]^ Standardized synthesis protocols and characterization criteria for large‐scale manufacture of NDC products are essential for NDC translation to the clinic but have been difficult to achieve due to e.g., numerous types of NDC (liposomes, polymers, metal NPs, etc.) used in preclinical and clinical studies, different experimental requirements and conditions, variable NP characteristic techniques and tools, lab staff across different laboratories. As a step forward, DeLoid et al.^[^ ^303^ ^]^ reported an integrated methodology to accurately determine the in vitro dosimetry of engineering nanomaterials (which is imperative to measure the biological outcomes), and Faria et al.^[^ ^304^ ^]^ proposed a “minimal information standard” including characterizations of NPs, biological effects, and experimental protocol details in nano‐bio studies. Nevertheless, continuous scientific efforts and standards along those lines will need to be set to make nano‐bio research more transparent, reproducible, and systematic.When moving nanotechnology towards clinical translation, it is imperative to know NP design–function relationship oriented not only from the chemistry and engineering perspective but also from biology and medicine perspective. The current need for NDC research is to address more clinically relevant challenges involving the understanding of disease pathophysiology and endotypes, as well as consideration of pharmacology. Chan and coworkers suggested a framework for designing in vivo NP delivery studies primarily consisting of clinical characterization of targeted diseases by imaging or histopathology analysis, computational simulation, and evaluation of drug performance and efficacy.^[^ ^11^ ^]^ A further recommendation by us for clinical‐guided NP technology would be taking more relevant ex vivo biomimetic physiological models derived from human tissues such as multiple cell culture systems, organ‐on‐a‐chip, and organotypic models (PCTS) into NDC delivery studies to fulfill biological gaps from the in vitro to in vivo studies. Those ex vivo model systems established either from human primary health or diseased cells or from a small piece of tissues exhibit many more biological and clinical features like multiple biological barriers, numerous cell types, spatial arrangement of tissue architecture, and complex microenvironments^[^ ^183^, ^224^, ^231^, ^235^ ^]^ over in vitro single cell line experiments, which has been systemically lacking in most nanomedicine research, probably due to the less involvement of clinicians and physicians as well as the unawareness of engineers and material scientists. Because of disease heterogeneity and genetic variations, precision NDC platforms might be only suitable for specific patient populations. Patient stratification combined with optimal delivery of customized NDC could largely enhance the opportunities of a successful NP delivery and treatment with greater therapeutic efficacy compared to NPs exploited for universe applications.Pursuing precision medicine in nanotechnology requires systematic understanding of nano‐bio interactions including NP dosimetry, pharmacokinetics, degradability, and ADME (absorption, distribution, metabolism, and excretion).^[^ ^305^ ^]^ This review thus discussed current state‐of‐the‐art microscopical technologies that allow for high‐resolution visualization and tracking of NP delivery and distribution in single cells, organs, and even organisms. Those methods would enable us to address several core questions in NP drug delivery research: where is the NPs' localization; are the NPs reaching the target sites with a satisfied amount, and which cell types accumulate most NPs? For example, tissue‐cleared LSFM imaging offers a holistic view of NP distribution within the whole organ or mouse body under cellular resolution that can't be reached by other in vivo fluorescent imaging or clinical PET or CT imaging modalities.^[^ ^239^, ^306^ ^]^ Artificial intelligence and machine learning algorithms should be further employed to comprehensively determine the nano‐bio interplay following the large NP dataset collected by new methodologies, e.g., applying machine learning‐based image analysis for quantification of NP delivered to primary tumors and micrometastases.^[^ ^299^ ^]^ Omics technologies (single cell or bulk tissue) have revolutionized many biomedical fields with new disease features and novel biomarkers being identified and are also applied to investigate the common responses and mechanism of cross‐species sensitivity to nanomaterial exposure using transcriptomic^[^ ^307^ ^]^ and to explore the heterogeneity of protein corona formed on polystyrene NPs by proteomics analysis with the liquid chromatography coupled to mass spectroscopy.^[^ ^308^ ^]^ In summary, we propose a personalized framework for NDC delivery towards clinical translation (**Figure** **16**) that encompasses disease diagnosis and biomarker identification through clinically used histopathology and advanced multi‐omics technology. Our approach emphasizes the development of novel designed NDC with full consideration of NP compositions and other physiochemical properties. Additionally, appropriate selection of effective delivery strategies and emerging pathophysiological models derived from human tissues, as well as application of advanced analytical (such as imaging and AI) tools are crucial for understanding nano‐bio interactions. Multidisciplinary collaboration among engineers, biologists, and clinicians is crucial to ensure the controlled use of NDC in biomedicine and facilitate the successful translation of nanomedicine into clinical practice.

## Conflict of Interest

The authors declare no conflict of interest.
